# Determiner spreading in Rukiga

**DOI:** 10.1515/ling-2021-0027

**Published:** 2023-09-06

**Authors:** Allen Asiimwe, Maria Kouneli, Jenneke van der Wal

**Affiliations:** Department of African Languages, School of Languages, Literature and Communication, Makerere University, Kampala, Uganda; Leiden University Centre for Linguistics, Leiden, The Netherlands; Department of Linguistics, Rutgers University, New Brunswick, USA

**Keywords:** augment, Bantu, determiner spreading, relative clause, restriction

## Abstract

Determiner spreading, the phenomenon whereby adnominal modifiers carry an ‘additional’ determiner, has been studied extensively for a variety of languages, most notably Greek, Semitic, and Scandinavian languages. Interestingly, the same phenomenon occurs in the Bantu language Rukiga. We show how the Rukiga augment is parallel to the Greek determiner in the context of modification, and how it triggers a restrictive reading when present on a larger class of modifiers than familiar so far: relative clauses, adjectives, possessives, and certain quantifiers. Considering its morphosyntactic and interpretational properties, we propose that the variation in the presence versus absence of the augment on modifiers is due to different underlying structures, applying an analysis of determiner spreading in terms of a reduced relative clause structure.

## 1 Introduction

The Bantu language Rukiga (Guthrie code JE14, spoken in western Uganda, often described together with mutually intelligible Runyankore JE13), shows noun class morphology throughout the noun phrase. The nouns themselves have a prefix and an augment (the initial vowel or pre-prefix) and adnominal modifiers show concord, as in (1). The choice of the augment vowel (*a*-, *e*-, *o*-) depends on the vowel of the prefix (see Asiimwe 2014; Hyman and Katamba 1993).1The same rules of vowel harmony that apply to Luganda apply to Rukiga.

(1)

**Table:** 

Rukiga

a.

**Table:** 

*e-bi-muri*	*(é-)bi-rúngi*
aug-8-flower	aug-8-beautiful
‘(the) beautiful flowers’

b.

**Table:** 

*a-ba-híngi*	*(á)-ba-mwe*
aug-2-chicken	aug-2-some
‘some (of the) farmers’

The examples in (1) show the pervasive noun class system and the optionality of the augment on (some) modifiers. Even though the presence of the augment on modifiers has been documented for many Bantu languages (e.g., de Blois 1970: 133–150; de Dreu 2008; Gambarage 2019 on Nata; Givón 1974 on Bemba; Halpert 2012, 2015 on Zulu; see also Nsuka-Nkutsi 1982; Van de Velde 2019), most existing work on the Bantu augment has focused on the semantic and syntactic factors conditioning its presence on the *noun* (see Halpert forthcoming; Van de Velde 2019 for recent overviews). Significantly less attention has been devoted to the distribution of the augment on *modifiers* (e.g., Gambarage 2019; Van de Velde 2019). In this article, we extend our understanding of the syntax and semantics of augments on modifiers by providing a detailed investigation of the behavior of the augment on modifiers in Rukiga, building on Asiimwe’s (2014) observation that the presence versus absence of the augment on modifiers in examples like (1) leads to a difference in interpretation.2See the Appendix and Asiimwe (2014) for an overview of the environments for the presence/absence of the augment on nouns. In the current article we concentrate on the augment on modifiers, not on nouns. Furthermore, we provide a syntactic analysis of the augment on modifiers, drawing inspiration from a striking parallel with the phenomenon of determiner spreading (or ‘polydefiniteness’) in Modern Greek, where (some) modifiers are optionally preceded by an ‘extra’ determiner, as illustrated in (2).

(2)

**Table:** 

Greek

a.

**Table:** 

*to*	*kokino*	*__*	*podilato*
the.n.sg	red.n.sg		bike.n.sg
‘the red bike’

b.

**Table:** 

*to*	*kokino*	*to*	*podilato*
the.n.sg	red.n.sg	the.n.sg	bike.n.sg
‘the red bike’			
(Kolliakou 2004)

For Greek, there is a general consensus that the interpretational contribution of the additional determiner is a restrictive reading of the modifier (e.g., Campos and Stavrou 2004; Kolliakou 2004; see also Tsiakmakis et al. 2021 for recent experimental work): Example (2a) with only a single determiner refers to a bike that happens to be red (non-restrictive), whereas Example (2b) with two determiners refers to the selection out of a set of various colored bikes that is red (restrictive).3Some work on Greek (including Campos and Stavrou 2004; Kolliakou 2004) uses the terms ‘intersective’ and ‘subsective’ to characterize the semantic difference described here. We use the term ‘restrictive’ in this article, as it is applicable to modifiers in general, and avoids discussion on (inherent) properties of adjectives as intersective or subsective.


In this article, we propose that the augment on modifiers in Rukiga functions just like the determiner in Greek determiner spreading in triggering a restrictive reading, and we show the underlying syntactic structures responsible for the variation in word order, morphology, and interpretation. We argue that the same theoretical machinery that has been proposed for Greek can also capture key properties of the phenomenon in Rukiga. Given the differences between the two languages, this is striking and can provide insights into the universal structure of DPs. The Rukiga data thus add to our knowledge of determiner spreading, and the structure of noun phrases more generally.

The remainder of the article is structured as follows: We provide a detailed investigation of the distribution of the augment on relative clauses in Section 2, and adjectives, possessives and quantifiers in Section 3. In Section 4, we propose our analysis: we show the similarities to Greek determiner spreading in 4.1, we discuss previous analyses of Greek determiner spreading in 4.2, and we provide underlying syntactic structures for Rukiga in 4.3. After commenting on the differences between the two languages in 4.4, we discuss in Section 5 the implications of our analysis and indicate avenues for further research.

## 2 Restrictive relatives in Rukiga

Both subject and object relative clauses in Rukiga can optionally take an augment. Object relatives are realized by an independent marker (based on the demonstrative), which may take an augment, as illustrated by the relative marker *(e-)yi* in (3b). There is no independent marker for subject relatives, as shown in (3c), and the optional augment is realized on the verb.

(3)

a.

**Table:** 

*W-aa-teek’*	*á-ka-ró*	*o-mu*	*n-yûngu.*
2pl.sm-n.pst-cook	aug-12-millet.bread	aug-18	9-pot
‘You have prepared *karo* in a pot.’

b.

**Table:** 

*e-n-yungw’*	*(* ***é*** *)-yí*	*wa-a-goya=mu*	*á-ká-ro*
aug-9-pot	aug-9rel.pro	2sg.sm-n.pst-mingle=18	aug-12-millet.bread
‘the pot that you prepared *karo* in’			[object relative]

c.

**Table:** 

e-n-yungw’	(**é**)-ya-a-teek’	á-ká-ro
aug-9-pot	aug-9rm-n.pst-cook	aug-12-millet.bread
‘the pot that was used to prepare the *karo*’		
lit. ‘the pot that has prepared the *karo*’		[subject relative]

According to Taylor (1985), the augment itself is a relative clause marker on subject relatives. However, since it can be omitted while retaining the relative meaning, it is clear that it cannot be regarded as a dedicated relative marker. Instead, the relative meaning is derived through tone pattern variation, as seen in (4). In (4a), the high tone on the subject marker and tense marker *yáá*- indicates a non-relative meaning; in (4b) on the other hand, the low tone on the prefixes yields a relative meaning.4Some other tenses in Rukiga use different segmental morphology to mark relatives. The remote past tense (in the affirmative) is marked by *ka*- in the non-relative clause (i). It changes to -*ire* in combination with *a*- in the relative counterpart (ii). We indicate the whole verb as relative by adding rel at the end of the gloss.

(i)

**Table:** 

*Wakamé*	*zi-ka-záar-a.*
10.rabbit	10sm-f.pst-give.birth-fv
‘(The) Rabbits gave birth.’

(ii)

**Table:** 

*wakamé*	*zaazíire*
wakame	za-a-zaar-íre
10.rabbit	10sm-pst-give.birth-pfv.rel
‘the rabbits that gave birth’

We gloss the former as sm and the latter as rm. See also Asiimwe (2019).

(4)

a.

**Table:** 

*Wakamé*	*y-áá-záar-a.*
9.rabbit	9sm-n.pst-give.birth-fv
‘A/the rabbit has given birth’.

b.

**Table:** 

*wakamé*	*y-aa-záar-a*
9.rabbit	9rm-n.pst-give.birth-fv
‘a/the rabbit which has given birth’

The question thus remains what function the presence/absence of the augment fulfills on relative clauses. Nsuka Nkutsi (1982: 105) indicates a difference in ‘emphasis’, and Taylor (1985: 22) refers not only to definiteness but also to the restrictive/non-restrictive difference in relative clauses. Our data show that the augment indeed distinguishes between restrictive and non-restrictive (appositive) relative clauses. Hence, we argue that the presence of the augment triggers a restrictive interpretation of the modified head noun, and when absent, a non-restrictive reading is obtained. The main difference in interpretation is illustrated in (5a) without the augment [−A] versus (5b) with the augment [+A].

(5)

a.

**Table:** 

*non-restrictive*			
*e-n-yungu*	***yí***	*wa-a-goy-a=mu*	*á-ká-ro*
aug-9-pot	9rel.pro	2sg.sm-n.pst-mingle-fv=18	aug-12-millet.bread
‘the pot, which you cooked *karo* in’			
(we already know which pot, there is one pot)

b.

**Table:** 

*restrictive*			
*e-n-yungw’*	***é-yí***	*wa-a-goy-a=mu*	*á-ká-ro*
aug-9-pot	aug-9rel.pro	2sg.sm-n.pst-mingle-fv=18	aug-12-millet.bread
‘the pot that you cooked *karo* in’ (not the other pot)

If our analysis is correct, we make the following predictions:

1. a [+A] (restrictive) relative clause should be incompatible with unique referents;
2. an answer to a ‘which’ question should require a [+A] relative clause;
3. a [+A] (restrictive) relative clause should only be felicitous when alternatives are available.

We present data and discussion for each prediction in turn.

First, unique referents are a useful test because they reject a restrictive reading. A restrictive relative clause selects a subset out of a set of alternatives, i.e., it restricts the reference of the head noun. If the referent is unique, however, there are no possible alternatives, and hence no restriction can be made. The sun, for example, is a unique entity: (outside of astronomy) we typically do not take alternative suns into consideration. It is therefore predicted to be incompatible with a restrictive relative clause, as no alternatives and hence no subset can be created. In Rukiga, indeed it is infelicitous to accompany it with a relative clause that carries an augment (6), as expected if the augment marks a restrictive relative clause. The presence of the augment on the relative clause was indicated by the speakers as meaning that there is more than one sun in the universe.

(6)

**Table:** 

*Ndeebir’ éízóób’ (* ^*#*^ *é)lirí hale.*			
n-reeb-ire	e-i-zooba	**e**-ri-ri	hare
1sg.sm-see-pfv	aug-5-sun	aug-5rm-be	far
‘I saw the sun, which is far.’			

Second, a typical environment for selection and restriction is the answer to a ‘which’ question as in (7a). This is because a ‘which’ question (unlike open wh questions) asks for a selection out of a given set, and the answer is expected to restrict the predicate to apply to a subset. Hence, the presence of the augment is strongly preferred on the relative clause in the answer in (7b), as it selects from among the different cloths. When the augment is missing, it means that there are no alternatives to select from, and the question-answer sequence is felt as being odd.

(7)

**Table:** 

(Context: At the market when looking at pieces of cloth in different colors)

a.

**Table:** 

*Orugóye nooyendá kugura ruuha?*			
o-ru-goye	ni-o-yend-a	ku-gura	ru-ha
aug-11-cloth	ipfv-2sg.sm-want-fv	15-buy	11-which
‘Which cloth do you want to buy?’

b.

**Table:** 

*Niinyendá kugur’ órugóy’* ^*#*^ *(ó)ruríkutukura*.^5^			
ni-n-end-a	ku-gura	o-ru-goye	**o**-ru-riku-tukur-a
ipfv-1sg.sm-want-fv	15-buy	aug-11-cloth	aug-11rm-ipfv-be.red-fv
‘I want to buy a/the red cloth.’, lit. ‘I want to buy a/the cloth that is red.’			

5 Many color terms in Rukiga are expressed as verbs, and their adnominal use is formally equal to a subject relative clause, as in (7b) for *-tukura* ‘be(come) red’. The same goes for verbs like *-osya* ‘be(come) hot’, *-fuka* ‘be(come) cold’ or *-terera* ‘be(come) silky’, as in (i) and (ii). See Asiimwe (2019). (i)

**Table:** 

*O-mw-énda*	*gw-á-terer-a.*
aug-3-cloth	3sm-n.pst-become.silky-fv
‘A/the cloth has become silky.’	
(ii)

**Table:** 

*o-mw-enda*	*gu-ríku-terer-a*
aug-3-cloth	3rm-prog-become.silky-fv
‘cloth which is silky/silky cloth’

Third, we tested whether [+A] relative clauses require alternatives through a picture-match test. We presented speakers with two pictures: The first showed only one church, which we indicated was built by the Bakiga; the second showed two churches, one built by the Bakiga and one by others. We asked the speakers to select the picture that matched the sentence, giving either sentence (8a) or (8b). Sentence (8a) with the [+A] relative clause was judged to refer to the picture showing two churches, thus restricting the relevant set of referents by excluding the other church. In contrast, sentence (8b) was judged more appropriate to describe the picture with only one church, confirming our analysis that the lack of the augment on the relative pronoun imposes a non-restrictive meaning.

(8)

a.

**Table:** 

*Nibakund’ ékanís’ éy’ Ábakíga baayômbekire.*			
ni-ba-kunda	e-kanisa	**e-i**	A-ba-kiga
ipfv-2sm-like	aug-9.church	aug-9.rel.pro	aug-2-kiga
*ba-a-yombek-ire*			
2rm-n.pst-build-pfv			
‘They like the church that the Bakiga constructed.’

b.

**Table:** 

*Nibakund’ ékanisa y’ Ábakíga baayômbekire.*				
ni-ba-kunda	e-kanisa	**i**	A-ba-kiga	ba-a-yombek-ire
ipfv-2sm-like	aug-church	9.rel.pro	aug-2-kiga	2rm-n.pst-build-pfv
‘They like the church, which the Bakiga constructed.’

From these tests, we conclude that the augment, when attached to relative clauses in Rukiga, imposes a restrictive interpretation of the relative clause while its absence implies a non-restrictive reading (appositive).6We note that in some noun classes, the initial vowel functions as an augment and a noun class prefix at the same time and therefore cannot be dropped. This is the case when the head noun of a subject clause belongs to class 1, 4, or 9, and also depends on tense of the verb. In those circumstances, tone still disambiguates between a restrictive and non-restrictive meaning, as shown in (i) and (ii).

(i)

**Table:** 

*Enyungw’ érimw’ ákahúng’ ekyáyosya.*			[restrictive]
e-n-yungu	e-ri=mu	a-ka-hunga	e-kya-yosy-a
aug-9-pot	9rm-be=18	aug-12-posho	9sm-still-be.hot-fv
‘The pot that has posho is still hot.’

(ii)

**Table:** 

*Enyungw’ erimw’ ákahúng’ ekyáyosya.*			[non-restrictive]
e-n-yungu	e-ri=mu	a-ka-hunga	e-kya-yosy-a
aug-9-pot	9rm-be=18	aug-12-posho	9sm-still-be.hot-fv
‘A/The pot, which has posho, is still hot.’

A reviewer points out that this situation “is the result of vowel hiatus resolution and that the initial vowel in such relative verb forms is a reflex of the augment, not the subject prefix. This strongly suggests that diachronically the augment is deleted in non-restrictive relative clauses rather than added in restrictive relative clauses. Where its segmental part has merged with the following class marker, only its tone is deleted.” We leave to further research the diachronic reanalysis that must have happened from the marked absence to the marked presence of the augment.


## 3 Restrictive modifiers

The same restrictive interpretation also determines the use of the augment on other modifiers, as we show in turn for adjectives, possessives, and quantifiers in this section. Brief discussion on the semantic or pragmatic nature of the restrictive interpretation follows in Section 5.

### 3.1 Adjectives

There are not many real adjectives in the Runyankore-Rukiga cluster generally. Taylor’s (1985: 174) list of ‘true’ adjectives has seventeen members and they are mainly in pairs appearing on opposite poles. Some of these adjectives are shown in (9).

(9)

a.

**Table:** 

*-hángo* ‘big’	-	*-kye* ‘small’

b.

**Table:** 

*-rungi* ‘good/beautiful’	-	*-bi* ‘ugly/bad

c.

**Table:** 

*-kúru* ‘old’	-	*-sya* ‘new’

d.

**Table:** 

*-raingwa* ‘tall’	-	*-gúfu* ‘short’

As mentioned in the introduction, adjectives in Rukiga display concord in noun class with the noun they modify, and they permit an optional augment. Taylor (1972, 1985 equated this augment to a definite marker. Hence, according to Taylor (1972: 74), the reference in (10a) is indefinite while in (10b) it is definite.

(10)

a.

**Table:** 

*o-mu-sháija*	*mu-rungi*
aug-1-man	1-good
‘a good man’

b.

**Table:** 

*o-mu-sháíj’*	***ó*** *-mu-rúngi*
aug-1-man	aug-1-good
‘the good man’	
(Taylor 1972: 74)

However, Asiimwe (2014: 263) shows that the augment may still be optionally present in typically indefinite environments, as in (11). The difference is thus not one of definiteness (see Asiimwe 2014, 2016 for further discussion on definiteness and specificity in Runyankore-Rukiga; see also Gambarage 2019 for a similar argument).

(11)

**Table:** 

*O-mu-sháíj’*	***(ó-)mu-rungí***	*n-oo-mw-ih-á*	*nkáhe?*
aug-1-man	aug-1-good	ipfv-2sg.sm-1om-get-fv	where
‘Where can you find a nice/good man?’			

Alternatively, the augment on adjectives in Rukiga has been associated with various (pragmatic) roles such as particularization, definiteness, specificity, focus and emphasis (Asiimwe 2014; Morris and Kirwan 1972; Taylor 1972, 1985). De Blois (1970: 134) notes that “In Nyoro, Nkore, Haya and Bemba only emphasized adjectives have the augment”. We build on these analyses, combining it with the insight from relative clauses in the previous section, and propose that the presence of the augment brings about a restrictive reading. Our expectations are that a [−A] adjective simply adds the attributive meaning (e.g., ‘big’), whereas the augmented adjective also triggers a set of alternatives to the adjective (e.g., ‘big, as opposed to small’).

**Figure 1:**
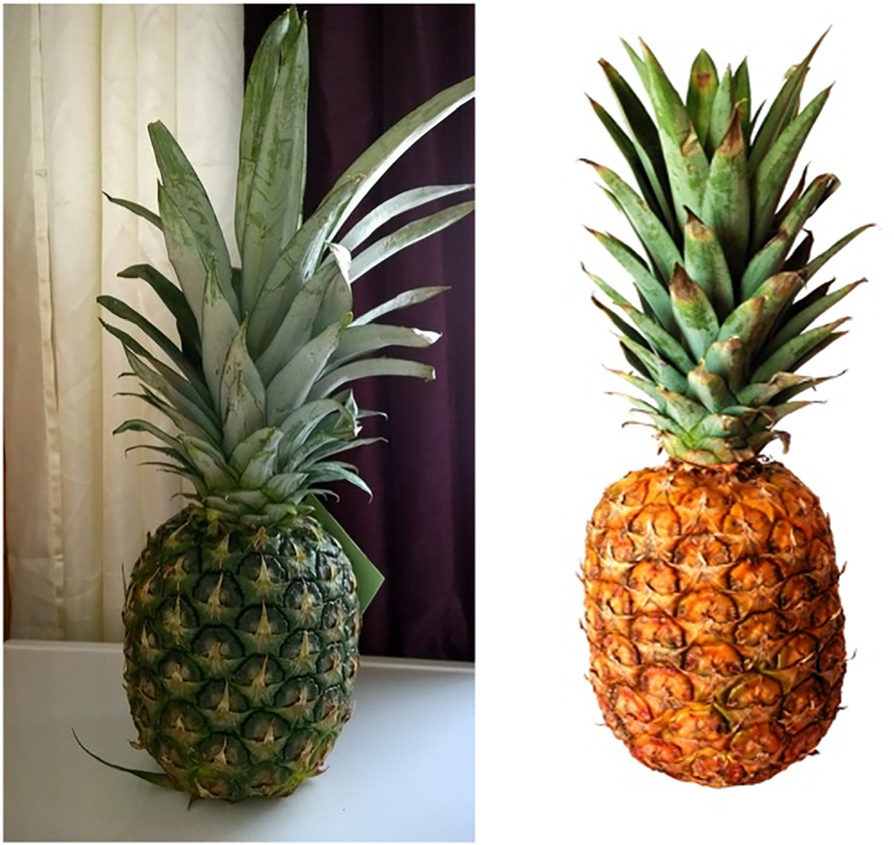
Set 1 ripe versus unripe pineapple (pictures via Pixabay, Wikimedia Commons, and Pixy).

**Figure 2:**
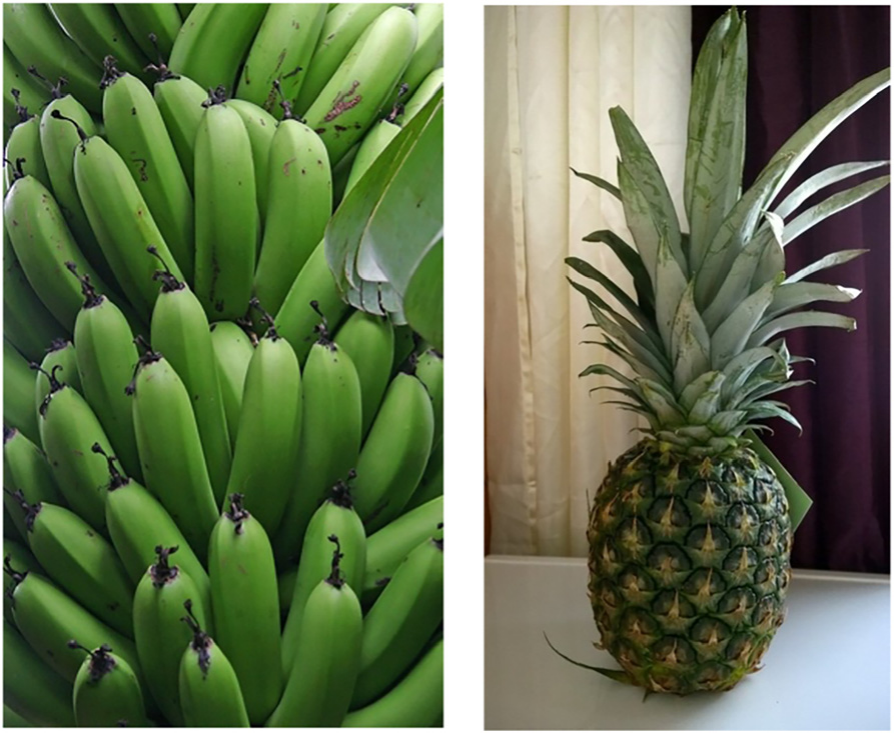
Set 2 unripe bananas versus unripe pineapple.

As with the relatives, here too we used pictures as a test to understand whether the [+A] adjective requires alternatives. Two sets of pictures were presented to the speakers (Figures 1 and 2). Set 1 contained pictures of a ripe pineapple and an unripe pineapple, keeping the referent the same but varying the adjectival quality. Set 2 contained pictures of an unripe pineapple and unripe bananas, contrasting the nominal referent while keeping the adjective constant. The speakers were then given a sentence with or without an augment on the adjective as in (12), and they were asked to indicate which of the two presented sets was appropriate for the given sentence.

We predicted that the [+A] adjective could only refer to Set 1 so that it selects a subset of pineapples (unripe), leaving the alternative (ripe pineapple). The [+A] adjective should not refer to the set containing the unripe pineapple and bananas, since no alternatives on the level of the adjective are present. These predictions were borne out: the speakers indicated that (12a), with an augment on the adjective, is appropriate for Set 1 with the ripe and unripe pineapple, whereas (12b), without the augment on the adjective, can be used for set 2. The pseudocleft in (12c) was offered by the speakers to highlight the contrast sought in the second set.

(12)

a.

**Table:** 

*Naagur’ énanáás’ émbísi.*			[+A]
n-aa-gur-a	e-nanaasi	**e-n-bisi**	
1sg.sm-n.pst-buy-fv	aug-9.pineapple	aug-9-unripe	
‘I have bought the unripe pineapple.’

b.

**Table:** 

*Naagur’ énanaasi mbísi.*			[−A]
n-aa-gur-a	e-nanaasi	**n-bisi**	
1sg.sm-n.pst-buy-fv	aug-9.pineapple	9-unripe	
‘I have bought an unripe pineapple.’

c.

**Table:** 

*Ekí nguzíre n’ énasaasi mbísi, etári minekye (mibísi).*					
e-ki	n-gur-ire	ni	e-nasaasi	n-bisi	e-ta-ri
aug-7.rel.pro	1sg.sm-buy-pfv	cop	aug-9.pineapple	9-unripe	9sm-neg-be
mi-nekye	mi-bisi				
4-banana	4-unripe				
‘What I bought is an unripe pineapple not (unripe) bananas.’

The same interpretation was also realized in a production task. This time the speakers were presented with Set 1 containing pictures of an old car and a new car, and Set 2 containing pictures of a car, a bus and an airplane, which were all new, and they were asked to describe each picture set. The speakers produced a [+A] adjective for Set 1, as in (13a); in contrast, a [−A] adjective was used for Set 2, as in (13b).

(13)

a.

**Table:** 

*E-mótok’ énsyá n’ éy’ ékika kya Toyóta kánd’ émótok’ énkúru n’ éy’ ékika kya BMW.*							
e-motok’	**e-n-sya**	ni	e-y-a	e-ki-ka	ky-a	Toyota	
aug-9.car	aug-9-new	cop	aug-9-conn	aug-7-make	7-conn	Toyota	

kandi	e-motoka	e-n-kuru	ni	e-y-a	e-ki-ka	ky-a	BMW
and	aug-9.car	aug-9-old	is	aug-9-conn	aug-7-make	7-conn	BMW

‘The new car is a Toyota and the old car is a BMW.’

b.

**Table:** 

*N-aa-reeb’*	*é-motoka*	***n-syá***	*hamwé*	*n’*	*é-nyonyí*	*n-sya.*
1sg.sm-n.pst-see	aug-9.car	9-new	and	and	aug-9.airplane	9-new
‘I saw a new car and a new airplane.’

A second test, again similar to the one used for relative clauses, is the answer to a ‘which’ question. This question targets one referent from a set of given alternatives, naturally (but not necessarily) eliminating those for which the proposition is not true. In answer (14b) to the question in (14a), the adjective with the augment selects big cups, entailing that the alternatives present in the context are small cups. When there are big and small cups in the context, it is not expected for the hearer to respond as in (14c) with a [−A] adnominal adjective – instead, (14c) is suitable for a question like ‘*What have you bought?*’. Similarly, a [+A] adjective would also be inappropriate when there are not only cups but different items to select from including, for example, plates, pots, spoons.

(14)

a.

**Table:** 

*E-bi-kópo*	*wa-a-gur-a*	*bi-iha ?*
aug-8-cup	2sg.sm-n.pst-buy-fv	8-which
‘Which cups have you bought?’

b.

**Table:** 

*N-aa-gur’*	*é-bi-kóp*	***é-bi-hángo***.
1sg.sm-n.pst-buy-fv	aug-8-cup	aug-8-big
‘I have bought the big cups.’

c.

**Table:** 

^*#*^ *N-aa-gur’*	*é-bi-kopo*	***bi-hángo***.
1sg.sm-n.pst-buy	aug-8-cup	8-big
‘I have bought big cups.’

A third test uses unique and typically non-unique referents, here illustrated with the terms for ‘son’ and ‘daughter’: as people typically have more than one child, reference to one child by their relative age is restrictive and takes a [+A] adjective, as in (15a). The [−A] form in (15b) is felt to be awkward, except perhaps in the circumstance where he has only one son, who happens to be young (but in this situation, one would simply refer to ‘his son’ rather than ‘his young son’). As expected, ‘mother’ and ‘father’ do not take [+A] modifiers, as one has only one (biological) mother and father.

(15)

a.

**Table:** 

*Yaamany’ éky’ ómutában’ ómut’ ákozíre.*				
a-aa-many-a	e-ki	o-mu-tabani	**o-mu-to**	a-kor-ire
1sm-n.pst-know-fv	aug-7.rel.pro	aug-1-son	aug-1-young	1sm-do-pfv
‘He found out what his youngest son did.’

b.

**Table:** 

*Yaamany’ éky’ ómutábani mut’ ákozíre.*				
a-aa-many-a	e-ki	o-mu-tabani	**mu-to**	a-kor-ire
1sm-n.pst-know-fv	aug-7.rel.pro	aug-1-son	1-young	1sm-do-pfv
*‘He found out what his youngest son did.’				
‘He found out what his (only) son, who is young, did.’

The term *omutabani* has a restricted meaning ‘son’. In contrast, *omuhara* can mean ‘daughter’ or ‘girl’, resulting in the interpretational difference between (16a) and (16b), depending on the presence or absence of the augment on the adjective.

(16)

a.

**Table:** 

*o-mu-hara*	***mu-kúru***
aug-1-girl	2-old
‘an old girl’

b.

**Table:** 

*o-mu-har’*	***ó-mu-kúru***
aug-1-girl	aug-2-old

‘the old girl’ (as opposed to the young one)	
‘oldest daughter’

Another illustration of the incompatibility between the augment and a unique referent is given in (17).

(17)

a.

**Table:** 

*Páápa*	***mu-kúru***	*y-aa-h’*	*ó-ru-bázo.*
1.pope	1-old	1sm-n.pst-give	aug-11-speech
‘The old Pope gave a speech.’

b.

**Table:** 

^*#*^ *Pááp’*	*ó-mu-kúru*	*y-aa-h’*	*ó-ru-bázo.*
1.pope	aug-1-old	1sm-n.pst-give	aug-11-speech
‘The old Pope gave a speech.’

Under the hypothesis that the augment induces a restrictive meaning, we also predict that non-intersective adjectives such as ‘former’ or ‘alleged’ should be incompatible with the augment (since they cannot receive a restrictive reading). We attempted to elicit such adjectives, but these notions are expressed via different constructions in Rukiga (e.g., a ‘president who is no longer there’ for ‘former president’, a ‘job that is able to be done’ for ‘possible job’), which are irrelevant to our point.

In summary, the presence of the augment on the adjective is an indication that there are alternatives to the selected referent and establishes a restrictive reading of the adjective.

### 3.2 Possessives

A possessive pronoun as a nominal modifier canonically follows the noun in Rukiga. In this post-nominal position, the possessive pronoun allows an optional augment, as in (18a). The connective -*a* ‘of’, which is a stand-alone morpheme connecting the noun and a DP possessor, also allows an optional augment, as shown in (18b).

(18)

a.

**Table:** 

*Ekikóp’ (e)kyé kikáátika.*		
e-ki-kopo	**(e-)ky-e**	ki-ka-atik-a
aug-7-cup	aug-7-poss.1	7sm-f.pst-break-fv
‘His/her cup broke.’

b.

**Table:** 

*Ekikóp’ (é)kya Émire kikáátika.*			
e-ki-kopo	**e-ky-a**	Emily	ki-ka-atik-a
aug-7-cup	aug-7-conn	1.Emily	7sm-f.pst-broke-fv
‘Emily’s cup broke.’

We again apply the diagnostics and come to the same conclusion: the augment triggers a restrictive reading. As before, the answer to a ‘which’ (or ‘whose’) question requires the use of a [+A] possessive, as shown in (19). The question in (19a) requires that one referent is selected from the rest. By answering (19b) it is understood that we do not take another car and it is inappropriate to answer the question with a [−A] possessive pronoun (19c). The interpretation of the augment attached to a connective as in (18b) above is the same: with the augment, cars of other people are presupposed and excluded.

(19)

a.

**Table:** 

*E-mótoka*	*tu-twár-e*	*e-ha?*
aug-9.car	1pl.sm-take-sbjv	9-which
‘Which car should we take?’

b.

**Table:** 

*Tu-twar-e*	*é-mótoka*	***é-y-ângye***.
1pl.sm-take-sbjv	aug-9.car	aug-9-poss.1sg
‘We take my car.’

c.

**Table:** 

^*#*^ *Tu-twar-e*	*é-mótoka*	***y-angye***.
1pl.sm-take-sbjv	aug-9.car	9-poss.1sg
‘We take my car.’

Further illustration comes from the minimal pairs in (20a)–(20b). Imagine I see someone searching everywhere in their bag and when I ask, they reply as (20a), which shows no subset on the basis of the possessor. But if the response were as in (20b), this implies that some item of the person searching is lost, whereas for other people, their items are not missing.

(20)

a.

**Table:** 

*Ekintú kyangye kibuzire.*		
e-ki-ntu	**ky-angye**	ki-bur-ire
aug-7-thing	7-poss.1sg	7sm-be.lost-pfv
‘My thing is missing.’

b.

**Table:** 

*Ekint’ ékyangyé kibuzire.*		
e-ki-ntu	**e-ky-angye**	ki-bur-ire
aug-7-thing	aug-7-poss.1sg	7sm-be.lost-pfv
‘It is (specifically) my thing that is missing.’

To illustrate again that the augment triggers alternatives on the level of the possessive, not the level of the DP, consider the contrast in (21). Both items – the hat and the scarf – belong to the speaker. Using a [+A] possessive is inappropriate here because there is no restriction to a subset on the level of the possessor. The [+A] possessive pronoun is only appropriate (and necessary) if there were hats and scarfs with different owners and it happens that for the rest, their hats and scarfs are dry, but mine are not.

(21)

**Table:** 

*Enkofíír’ (* ^*#*^ *é)yangyé teyomire. Kándi na sikááfu (* ^*#*^ *e)yangyé nayó teyomire.*					
e-n-kofííra	**(** ^**#**^ **e)-y-angye**	ti-e-om-ire.	kándi	na	sikááfu
aug-9-hat	aug-9-poss.1sg	neg-9sm-dry-pfv	and	and	9.scarf

***(*** ^***#***^ ***e)-y-angye***	*na-y-ó*	*ti-e-om-ire*			
aug-9-poss.1sg	and-9-pro	neg-9sm-dry-pfv			
‘My hat is not dry. And even my scarf is not dry.’					

### 3.3 Quantifiers

The nominal modifiers -*kye* ‘few, little’ and -*ingi* ‘many, several, much’, like the relatives, adjectives, and possessives above, permit an optional augment. Both of these quantifiers refer to a non-restrictive number or quantity when used [−A], translated as ‘few/little’ and ‘many/much’, respectively. With the presence of the augment, -*ingi* is rendered as ‘most of’ or ‘majority of’, as shown in the minimal pair in (22).

(22)

a.

**Table:** 

*Enju nyîngi zitiir’ érángi.*			
e-n-ju	**ny-ingi**	zi-teer-ire	e-rangi
aug-10-house	10-many	10sm-beat-pfv	aug-9.color
‘Many house are painted.’

b.

**Table:** 

*Enjw’ ényîngi zitiir’ érángi.*			
e-n-ju	**e-ny-ingi**	zi-teer-ire	e-rangi
aug-10-house	aug-10-many	10sm-beat-pfv	aug-9.color
‘The majority/most of the houses are painted.’

Here too, we believe that the augment restricts the interpretation to a subset of the noun, as it does in restrictive relative clauses, adjectives, and possessives. The presence of the augment selects the larger subset of houses, i.e., the subset of houses that are many, leaving a smaller subset of houses that are few, so to speak. This restriction is more idiomatically rendered as ‘most of’.

As with the other modifiers, in the answer to the ‘which’ question in (23a), the [−A] quantifier -*ngi* or -*kye* is not appropriate (23b), and the [+A] form of the quantifier is required. The presence of the augment here indicates that there were containers of porridge such that one contained more porridge than another one or than the rest.

(23)

a.

**Table:** 

*O-bu-shera*	*wa-a-nyw-a*	*bu-uha?*
aug-14-porridge	2sg.sm-n.pst-drink-fv	14-which
‘Which porridge have you eaten?’

b.

**Table:** 

^*#*^ *O-bu-shera*	***bw-íngi***	*ni-bw-ó*	*ná-á-nyw-a.*
aug-14-porridge	14-much	cop-14-rel.pro	1sg.sm-n.pst-drink-fv
‘I have taken a lot of porridge.’

c.

**Table:** 

*O-bu-sher’*	***ó-bw-íngi***	*ni-bw-ó*	*ná-á-nyw-a.*
aug-14-porridge	aug-14-much	cop-14-rel.pro	1sg.sm-n.pst-drink-fv
‘I have taken the porridge that was a lot.’

A similar restriction of the referent is found when -*kye* ‘few, little’ is used with mass nouns. The [−A] form of the quantifier in (24a) just indicates a small quantity, whereas the [+A] form in (24b) indicates a countable referent, that is, the quantifier applies to a restricted referent, as opposed to alternative quantities.

(24)

a.

**Table:** 

*a-ma-izi*	***ma-kye***
aug-6-water	6-few
‘little water’

b.

**Table:** 

*a-ma-íz’*	***á-má-kye***
aug-6-water	aug-6-few

‘few (bottles of) water’	
‘a container with the smallest quantity of water’

A third quantifier, -*mwe* ‘some’, seems to work just the same: -*mwe* with an augment selects some and excludes others in a given set, as in (25b). The augment seems to add a stronger implication that the selected set is a proper subset (though see Section 5 for discussion on the semantics and pragmatics of restriction). This quantifier can also be used to mean ‘certain’ when used without the augment, although this meaning is preferably expressed by a presentational construction with *ha-ine* (16sm-have) or *ha-ri=ho* (16sm-be=16) ‘there is’.

(25)

a.

**Table:** 

*Amaju gamwé gatiir’ érángi.*			
a-ma-ju	**ga-mwe**	ga-teer-ire	e-rangi
aug-6-house	6-some	6sm-beat-pfv	aug-9.color
‘Some/certain houses are painted.’

b.

**Table:** 

*Amajw’ ágamwé gatiir’ érángi.*			
a-ma-ju	**a-ga-mwe**	ga-teer-ire	e-rangi
aug-6-house	aug-6-some	6sm-beat-pfv	aug-9.color
‘Some of the houses are painted.’

If our hypothesis is correct that the presence of the augment on modifiers requires a set of alternatives, we can make two further predictions for quantifiers. The first is that the augment should be incompatible with a universal quantifier -*ona* ‘all’, since this does not allow for alternatives. This is borne out, as shown in (26).

(26)

**Table:** 

*E-bi-hunyirá*	(* ***e*** *-)* ***by-óna***	*bi-in’*	*á-ma-gezi*	*ma-íngi.*
aug-8-owl	aug-8-all	8sm-have	aug-6-wisdom	6-much
‘All owls are very wise.’				

A second prediction is that the modifier -*ndi* ‘other’ actually requires the use of the augment, since it entails the presence of alternatives: if there is an ‘other’, then there must be a ‘one’. This too is borne out, as -*ndi* always appears with the augment (27).

(27)

**Table:** 

*O-mu*	*ka-shéésh’*	*( ***é*** *-)* ***zí-ndi***	*nyamaishwá*
aug-18	12-morning	aug-10-other	10.animal

kú	zi-iz-ire	ku-kóra…	
when	10sm-come-pfv	15-work	

‘In the morning when (the) other animals came to work…’			

### 3.4 Summary

In summary, we have provided evidence for the claim that the augment, when present on adnominal adjectives, possessives and the quantifiers *-ingi/-kye/-mwe* indicates that the speaker aims to alert the hearer that the intended referent is selected out of a set containing alternatives, only a restricted subset of which is characterized by the modifier. In the absence of an augment on the other hand, there are no alternatives implied and hence no restriction. The semantic impact of augments on Rukiga modifiers also shows that this phenomenon cannot be analyzed as ‘case concord’, as Halpert (2015) proposes for Zulu augments.

The possibilities and interpretations for the different modifiers are summarized in Table 1.

**Table 1:** Possibilities and interpretation of Rukiga modifiers with and without augment.

	−A	+A
*relative*	Non-restrictive	Restrictive
*adjective*	Neutral	Restrictive
*possessive*	Neutral	Restrictive
*many/few/some*	Quantity	Subsection of quantity
*all*	✓	✗
*other*	✗	✓

For completeness, we note that adnominal numerals in Rukiga do not take an augment in the presence of an overt noun, as illustrated in (28a). In pronominal uses (i.e., contexts without an overt noun), as in (28b), a [−A] numeral is preferred. In subject position, in the absence of an explicit head noun, a [−A] is as acceptable as a [+A] numeral, shown in (28c). Numerals are thus different from other augment-allowing nominal modifiers, which take an obligatory augment when in an elliptical structure in subject position.

(28)

a.

**Table:** 

*A-ba-híigi*	*ba-k-ombek’*	*ó-bu-siisira*	(*o-)bú-shatu.
aug-2-hunter	2sm-f.pst-build	aug-14-hut	aug-14-three
‘(The) hunters built three huts.’

b.

**Table:** 

*A-ba-híigi*	*ba-k-ombek’*	*(* ^*?*^ *ó-)bú-shatu.*
aug-2-hunter	2sm-f.pst-build	aug-14-three
‘(The) hunters built (the) three (huts).’

c.

**Table:** 

*(O-)bú-shatu*	*bu-k-ombek-w‘*	*á-ba-híigi.*
aug-14-three	14sm-f.pst-build-pass	aug-2-hunter
‘The three were built by (the) hunters.’

As for demonstratives, whether used pronominally or adnominally, they never take an augment. The initial element of the demonstrative is not an augment but the core demonstrative morpheme (Asiimwe 2014, 2016).

## 4 Rukiga augmented modifiers and Greek determiner spreading

The distribution of the augment on modifiers in Rukiga shows striking similarities to the better-studied phenomenon of Determiner Spreading (DS) in Greek, also called ‘polydefiniteness’ in the literature (e.g., Alexiadou and Wilder 1998; Androutsopoulou 1995, 2001; Campos and Stavrou 2004; Kolliakou 2004; Lekakou and Szendrői 2012; Panagiotidis and Marinis 2011; Velegrakis 2011). In this phenomenon, a single DP contains multiple instances of the definite determiner in the context of (usually) adjectival modification. This is illustrated in (29), where either one (as in 29a) or two (as in 29b) definite articles may be present in a simple definite DP with a noun and an adjective.

(29)

a.

**Table:** 

**to**	kokino	podilato
the.n.sg	red.n.sg	bike.n.sg
‘the red bike’

b.

**Table:** 

**to**	kokino	**to**	podilato
the.n.sg	red.n.sg	the.n.sg	bike.n.sg
‘the red bike’			
(Kolliakou 2004: 264)

DS is attested in a range of languages from different language families (see Alexiadou 2014; Kouneli 2019; Lekakou 2017 for overviews), but there is significant cross-linguistic variation; this is why Alexiadou (2014) concludes that DS does not constitute a unified phenomenon across languages, and she proposes three possible types of DS, each one associated with a different syntactic structure (see Section 5 for further discussion of Alexiadou’s typology). The properties of DS in Greek exemplify one of these types, and the goal of this section is to show that the distribution of the augment on nominal modifiers in Rukiga shares most of these properties. We will thus argue that the augment on Rukiga modifiers should be analyzed on a par with determiner spreading in Greek. In Section 4.1, we discuss the similarities between Greek DS and Rukiga augmented modifiers that motivate this claim. In Section 4.2, we present two previous analyses of Greek DS and discuss the predictions they make for Rukiga, and in Section 4.3, we present our complete analysis of the Rukiga data. The differences between the two languages are discussed in Section 4.4.

It should be made clear that ‘spreading’ is simply the descriptive term used in the literature (especially on Greek) to refer to the phenomenon of multiple determiners in a single DP in the context of modification; the term does not refer to any theoretical mechanism, and in fact various theoretical devices have been proposed in the literature (see Section 4.2). We do not suggest that the determiner ‘spreads’ in either Greek or Rukiga, but use DS to refer to the optional presence of a determiner on an adnominal modifier. Indeed, the fact that the augment may be present on a modifier in an environment where the determiner on the noun must be null (see Section 4.1.1), as in *Kato (o)muraingwa* ‘tall Kato’, shows that DS is not about ‘spreading’ of an overt determiner.

### 4.1 Greek DS and Rukiga augments

Greek DS displays a number of syntactic and semantic properties that bear significant similarities to the distribution of the Rukiga augment on modifiers outlined in the previous sections (see also Gambarage 2019: 138–141 for mention of a similar observation for Nata, Bantu E45). We discuss and illustrate these in turn, and we return to the differences in Section 4.4.

#### 4.1.1 The article in Greek and the augment in Rukiga are both determiners in D

We start with the basic question of the nature of the element that participates in spreading in the two languages: the definite article in Greek and the augment in Rukiga. In the case of Greek, it is relatively uncontroversial that the article is in D. We argue that the augment in Rukiga is also associated with D.7In generative syntax, D is assumed to be the functional category heading the noun phrase (Abney 1987 and subsequent work). It is a syntactic category and is not necessarily associated with the same semantics in all languages.


The augment on the noun has been analyzed as a determiner in D for many other Bantu languages (see De Dreu 2008; Gambarage 2013, 2019; Visser 2008 for specific languages; and Halpert forthcoming for a Bantu-wide overview of the augment). In Rukiga, we find indirect evidence that the augment is in D: where a form with an augment exists,8Note that not all nouns in Rukiga have a form with an augment, for example, proper names, nouns in class 1a, or derivations in *nya*- simply do not exist with an augment. Nevertheless, we assume that such nouns may still project D but not show this in morphology. Their behavior as arguments (not predicates) and their status as “inherently referential” (Van de Velde 2019: 250) form evidence to analyze them as DPs in such environments, as Longobardi (1994) also argues. the presence of the augment is ungrammatical in exactly those contexts where we would expect an NP, and not a DP, in other languages. We therefore find [−A] nouns as vocatives (30) (see Longobardi 1994 on DP arguments vs. NP non-arguments), and in compounds (31) – see the appendix for an overview of the augment on nouns.

(30)

a.

**Table:** 

*Bo-ojo!*	(cf. *a-b-oojo* ‘boys’)
2-boys	
‘Boys!’

b.

**Table:** 

*Aboojo!

(31)

a.

**Table:** 

*a-ka-cwá-n-koni*	(cf. *e-n-koni* ‘walking stick’)
aug-12-break-9-walking.stick	
(name of a nocturnal bird that whistles)

b.

**Table:** 

*a-ka-cwá-e-n-koni

Furthermore, (32a) and (32b) show that the augment is in complementary distribution with the prenominal quantifier *buri* ‘every’ and the question word *ki* ‘which’. These are standardly analyzed as quantificational and interrogative determiners, respectively, which cross-linguistically select for NP. Note that it is the hierarchical structure that matters here, and not the linear order.

(32)

a.

**Table:** 

*buri*	(*o-)mw-ojo
every	aug-1-boy
‘every boy’

b.

**Table:** 

(*E)-ki-róótó	*ki?*
aug-7-dream	which
‘Which dream?’

Given that *buri* ‘every’ and *ki* ‘which’ could be analyzed as D heads, an anonymous reviewer asks why those elements cannot participate in spreading. We do not currently have a satisfactory answer to this question, but we note that it forms part of a larger debate about which determiners can spread and why. For example, according to the overviews in both Alexiadou (2014) and Lekakou (2017), indefinite determiner spreading is much less frequently attested cross-linguistically (note that at least *ki* ‘which’ could be thought of as an indefinite determiner). We refer the interested reader to these works for an overview of the explanations that have been offered in the literature for the restrictions on which determiners may participate in spreading.

What is clear, is that demonstratives are not in the same structural position as the augment, as the two can cooccur – optionally with a prenominal demonstrative (33a) and obligatorily with a postnominal demonstrative (33b). We will not delve further into this difference in word order here, and refer to Asiimwe (2016, to appear) for discussion of the augment and demonstratives.

(33)

a.

**Table:** 

*e-gy-o*	*(e-)m-baraasi*
dem-9-med	aug-9-horse
‘that horse’

b.

**Table:** 

*(e-)m-baraasi	*e-gy-o*
aug-9-horse	dem-9-med

Unlike some other Bantu languages (De Blois 1970), non-verbal predication does not require the absence of the augment in Rukiga. Instead, Rukiga always uses the copula *ni* – compare Example (35) with Lusoga in (34). Rukiga does distinguish between DP predicates (with augment, (35a), and adjectival predicates (without augment, (35b)).

(34)

**Table:** 

Lusoga

a.

**Table:** 

éé-nkokó
‘chicken’

b.

**Table:** 

ń-kokó
‘it is a chicken’
(JE16; data Van der Wal fieldwork)

(35)

**Table:** 

Rukiga

a.

**Table:** 

(What does Kato do for a living?)		
*Kató*	*n’*	*ó-mu-shomésa.*
1.Kato	cop	aug-1-teacher
‘Kato is a teacher.’

b.

**Table:** 

*Kató*	*ní*	*mu-raingwa.*
1.Kato	cop	1-tall
‘Kato is tall.’

If the Greek article and the Rukiga augment are both determiners, we can understand another structural parallel between the two languages: the fact that pronominal modifiers (here illustrated for adjectives) require the presence of the article/augment, deriving a referential DP.9Possibly with the exception of numerals, as discussed above.

(36)

**Table:** 

Greek		
*(to)	*petrino*	*(spiti)*
the.n.sg	stone.n.sg	(house.n.sg)
‘the stone one’		
(Lekakou and Szendrői 2012: 120)

(37)

**Table:** 

Rukiga				
*(e-)bi-hángo	/	*(a-)ba-hángo	/	*(e-)mi-hángo
aug-8-big		aug-2-big		aug-4-big
‘the big ones’				

We therefore conclude that the augment in Rukiga is best analyzed as a determiner associated with D. It is clear, however, that the augment in Rukiga cannot be analyzed as a *definite* determiner, seeing as it can also be used with an indefinite interpretation (see (11)) above and further argumentation in Asiimwe 2014, 2016). This might at first glance seem very different from the situation in Greek, where the determiner in question is the ‘definite article’. However, there is a debate in the literature about the analysis of the article in Greek. Many scholars have argued that the article is in D, but the locus of definiteness is in a separate head (e.g., Def) instead (Lekakou and Szendrői 2012, among others). To illustrate, the ‘definite’ article is in some cases possible in clearly indefinite contexts, such as the modification of the indefinite pronoun *kati* ‘something’ in (38).

(38)

**Table:** 

*kati*	*to*	*dhiaforetiko*
something	the	different
‘something different’		

Thus, even though there are certainly differences in the exact behavior of the augment and the article in Rukiga and Greek respectively (see also Section 4.4), both elements are determiners in D that are not (always) associated with definite semantics.

#### 4.1.2 The additional determiner is obligatory in non-canonical word orders in both languages

We turn next to the ordering possibilities between the noun and its modifiers in the two languages. In the absence of additional determiners, the unmarked order between the noun and the adjective is Noun – Adjective in Rukiga, but Adjective – Noun in Greek, as shown in (39) and (40).10Word order possibilities in Greek depend on definiteness: while the adjective must precede the noun in definite DPs (in the absence of DS), it can either precede or follow the noun in indefinite DPs (Alexiadou and Wilder 1998). We focus here on definite DPs, since DS is not possible with indefinite determiners in Greek.In Rukiga, all modifiers are post-nominal by default, apart from the invariable quantifier *buri* ‘every’ which is always prenominal; demonstratives, possessives, relative clauses, and some quantifiers may precede or follow the noun (Asiimwe to appear; Van de Velde 2005). In Greek, the position of the modifier depends on its category: adjectives, numerals, and quantifiers are pre-nominal, but relative clauses and possessors are post-nominal. We focus here on adjectives since DS is limited to those (and numerals) in Greek. The determiner precedes the noun in both languages.

(39)

**Table:** 

Greek

a.

**Table:** 

to	kokino	podilato
the.n.sg	red.n.sg	bike.n.sg

b.

**Table:** 

*to	podilato	kokino
the.n.sg	bike.n.sg	red.n.sg
‘the red bike’		
(Kolliakou 2004: 264)

(40)

**Table:** 

Rukiga

a.

**Table:** 

*e-bi-muri*	*bi-hángo*
aug-8-flowers	8-big
‘big flowers’

b.

**Table:** 

*bi-hángo	*e-bi-muri*
8-big	aug-8-flowers
‘big flowers’	
(Asiimwe 2014: 257)

DS is optional (modulo the semantic/pragmatic effects discussed in this article) in this word order in both languages. What is interesting, however, is that DS is obligatory in non-canonical orders: with post-nominal modifiers in Greek and pre-nominal modifiers in Rukiga, shown in (41) and (42). In other words, DS gives rise to additional ordering possibilities within the DP. (Note that these orders are not simply two nominal elements, e.g., ‘the red one, the bike’. We return to the subtle prosodic and interpretational differences in Section 4.3).

(41)

**Table:** 

Greek			
*(to)	*podilato*	*(to)	*kokino*
the.n.sg	bike.n.sg	the.n.sg	red.n.sg
‘the red bike’

(42)

**Table:** 

Rukiga	
*(e)-bi-hángo	*(é)-bi-muri
aug-8-big	aug-8-flowers
‘big flowers’	
(Asiimwe 2014: 257)	

#### 4.1.3 The determiner can be present with multiple modifiers

In both Greek and Rukiga, each additional modifier may in principle be preceded by a determiner, resulting in multiple instances of the determiner in the DP. This is illustrated in (43) for Greek and in (44) for Rukiga. The possible combinations of modifiers with and without determiners are discussed in Section 4.3.2.

(43)

**Table:** 

Greek					
*to*	*podilato*	*to*	*kokino*	*to*	*ghrighoro*
the.n.sg	bike.n.sg	the.n.sg	red.n.sg	the.n.sg	fast.n.sg
‘the red fast bike’

(44)

**Table:** 

Rukiga			
*e-bi-muri*	*(é-)bi-rungi*	*(é-)bi-hángo*	*(é-)bí-ngi*
aug-8-flower	aug-8-beautiful	aug-8-big	aug-8-many
‘many (of the) big beautiful flowers’			

#### 4.1.4 The additional determiner is associated with a restrictive interpretation in both languages

The semantic interpretation of [+A] modifiers in Rukiga was discussed extensively in Section 3. Our conclusion was that the presence of the augment on modifiers leads to a restrictive interpretation. As already hinted at in the introduction, the same semantic effect has been documented for Greek DS.

Alexiadou and Wilder (1998) describe the interpretational difference between the absence and presence of the second article in Greek as that between non-restrictive and restrictive adjectives, respectively. We repeat Example (2) here as (45): (45a) without DS refers to a bike, which just happens to be red, whereas (45b) refers to the selected red bike out of a set of bikes in various colors.

(45)

**Table:** 

Greek

a.

**Table:** 

*to*	*kokino*	*__*	*podilato*
the.n.sg	red.n.sg		bike.n.sg
‘the red bike’

b.

**Table:** 

*to*	*kokino*	*to*	*podilato*
the.n.sg	red.n.sg	the.n.sg	bike.n.sg
‘the *red* bike’			
(Kolliakou 2004)

The restrictive reading is confirmed by the infelicity of the double determiner with a pleonastic adjective, as in (46): since all cobras are poisonous, it is impossible to establish a restrictive reading (that is, a set of alternatives is not available), hence ruling out the use of the second determiner.

(46)

**Table:** 

Greek

a.

**Table:** 

*Idame*	*tis*	*dilitiriodis*	*__*	*kobres.*
saw.1pl	the.f.pl	poisonous.f.pl		cobras.f.pl
‘We saw the poisonous cobras.’

b.

**Table:** 

^*#*^ *Idame*	*tis*	*dilitiriodis*	*tis*	*kobres.*
saw.1pl	the.f.pl	poisonous.f.pl	the.f.pl	cobras.f.pl
(‘We saw the *poisonous* cobras.’)				
(Kolliakou 2004: 274)

Replicating the example in Rukiga results in the same for the relative clause ‘that has poison’: the augmented form is not acceptable.

(47)

a.

**Table:** 

*e-n-cwera*	*zí-íne*	*o-bu-shegu*
aug-10-cobra	10rm-have	aug-14-poison
‘poisonous cobras’, lit. ‘cobras that have poison’

b.

**Table:** 

^*#*^ *e-n-cwera*	*é-zi-ine*	*ó-bu-shegu*
aug-10-cobra	10rm-have	aug-14-poison
int. ‘*poisonous* cobras’

We therefore conclude that both DS in Greek and augmented modifiers in Rukiga give rise to restrictive interpretations of nominal modifiers.11There are certain apparent counterexamples to this claim for Greek. For example, in the following example (for which we thank Georg Höhn), there is no reference to a set of alternatives, and only one wife exists.

(i)

**Table:** 

*i*	*kali*	*mu*	*i*	*jineka*
the.f.sg	good.f.sg	my	the.f.sg	woman
‘my good/dear wife’

However, these examples show certain unique properties, and Alexiadou (2014: 40–42) argues that these constructions are different from the phenomenon of DS in the language. To name some of the characteristics of these constructions: first, the adjective often has an idiomatic interpretation (e.g. ‘dear’ in (i) above). Second, there is an ‘emotive’ aspect to all these examples, and they only occur in the Adjective – Noun order; the reverse order does force a restrictive interpretation. However, we also observe a difference between the two languages: Whereas an augment is possible on almost any nominal modifier (including full relative clauses) in Rukiga, DS in Greek is restricted to adjectives, numerals, and marginally some quantifiers (e.g., *pola* ‘many’). We return to this difference in Sections 4.3 and 4.4.

#### 4.1.5 The additional determiner is only possible with indirect modification adjectives in both languages

Research on the syntax and semantics of adjectives has revealed that there are two broad types of adjectives, called *direct* and *indirect modification* adjectives in Cinque’s (2010) terminology (which is itself borrowed from Sproat and Shih 1988). The distinction has been discussed in many studies (e.g., Alexiadou et al. 2007; Larson 1995, 1998; Sproat and Shih 1988), but Cinque (2010) includes the most comprehensive summary of all syntactic and semantic differences between the two types of adjectives. An example often used in the literature to illustrate the distinction is (48) below, where the differences between the two types of adjectives are correlated with the relative placement of the adjective and the noun in English. When *visible* follows the noun, as in (48b), it can only receive a restrictive interpretation: there must exist stars that are not visible at the time of utterance of such a phrase. An adjective with these properties is an indirect modification adjective. When the adjective precedes the noun, however, a non-restrictive interpretation is also possible; furthermore, the stars’ visibility can be independent of the time of utterance (e.g., we could be speaking of stars that are always visible in the night sky).12See Cinque (2010) for more details on the temporal properties of the two types of adjectives; the terms *stage-level* and *individual-level* interpretations are often used in this context. If the adjective receives this interpretation, then it is a direct modification adjective.13It should be noted that in this case, the adjective *visible* is ambiguous between the two interpretations when in pre-nominal position, as in (45a). In Cinque (2010), the two possible interpretations will correspond to two different syntactic structures. What this means is that even though post-nominal adjectives in English are unambiguously indirect modification adjectives, pre-nominal ones could be either direct or indirect modification adjectives, depending on their underlying structure (which can be obscured by subsequent movement).

(48)

a.

**Table:** 

*the visible stars*

b.

**Table:** 

*the stars visible*

While we do not discuss the details of the available theoretical treatments of the distinction, we note that almost all previous studies agree that indirect modification adjectives should be analyzed as reduced relative clauses; most of the differences between analyses lie in the technical implementation of this idea.

One of Cinque’s (2010) diagnostics refers to the restrictive versus non-restrictive distinction that we often find in nominal modification. According to him, indirect modification adjectives consistently have a restrictive interpretation, while direct modification adjectives have a non-restrictive interpretation. As has already been discussed at length, the presence of an augment on the modifier always requires a restrictive interpretation, and in the previous section we saw that this observation also holds for DS in Greek. This diagnostic, therefore, shows that only indirect modification adjectives participate in the phenomenon.

Another diagnostic distinguishing between the two types of adjectives is found in the ordering possibilities that we find among multiple adjectives. While direct modification adjectives are subject to strict ordering restrictions cross-linguistically (e.g., size < color), indirect modification adjectives display free word order. We have already seen in Section 4.1.2 that the presence of additional determiners in both Greek and Rukiga give rise to additional ordering possibilities in the DP, indicating that the adjectives participating in the phenomenon are not of the direct modification type.

A final observation that has long been known about Greek DS is that it is only possible with adjectives that can also appear in predicative position; in other words, it is ungrammatical with adjectives that can only appear in attributive position, such as *proin* ‘former’, illustrated in (49).14The incompatibility of DS with non-predicative adjectives in Greek has been challenged (e.g. Cinque 2010; Lekakou and Szendröi 2007). It has been observed, however, that for those speakers who allow DS with non-predicative adjectives, the adjective has in those cases been coerced into a restrictive modifier (see Alexiadou 2014: 33, footnote 4 for discussion).

(49)

**Table:** 

Greek

a.

**Table:** 

*O	*proedhros*	*ine*	*proin.*
the.m.sg	president.m.sg	is	former
‘*The president is former.’

b.

**Table:** 

*o*	proin (*o)	*proedhros*
the.m.sg	former the.m.sg	president.m.sg
‘the former president’

Since purely attributive adjectives (such as *former*, *main*, *alleged* etc.) cannot appear in predicative position, they resist a reduced relative analysis and are always classified as direct modification adjectives in Cinque’s (2010) terminology. The ungrammaticality of DS with these adjectives indicates a connection to indirect modification and relative clause structures, which has played a central role in certain analyses of the phenomenon (e.g., Alexiadou and Wilder 1998), as we will see in the next section.

These facts about Greek are difficult to replicate in Rukiga, since all adjectives can also appear in predicative position and notions such as *former* or *alleged* are expressed by other means, as indicated in Section 3.1 above. Foreshadowing our analysis in Section 4.3, however, the fact that adjectives can always be used predicatively, in addition to the availability of additional augments on full relative clauses, indicates that a relative clause analysis along the lines of Alexiadou and Wilder (1998) is plausible for Rukiga, a point we will discuss in detail in the next sections.

To sum up, we have shown that there are a number of similarities between DS in Greek and ‘augment spreading’ in Rukiga (despite significant differences in DP syntax in the two languages), which suggests that both should be treated as variants of the same phenomenon. We turn next to previous analyses of DS in Greek, focusing on the predictions they make for the Rukiga data.

### 4.2 Approaches to Greek DS

There is a large number of analyses of Greek DS, which we cannot do justice to in this article (see Alexiadou 2014; Lekakou 2017; Tsiakmakis et al. 2021 for overviews). Instead, we focus on two analyses, inspired by the properties that Rukiga and Greek have in common. The first is Lekakou and Szendrői’s (2012) analysis of DS as close apposition. While this analysis explains many of the observed properties, it also makes some wrong predictions, as discussed in Section 4.2.1. We therefore continue to a second analysis that uses a reduced relative clause structure (Alexiadou 2014; Alexiadou and Wilder 1998) in Section 4.2.2.

#### 4.2.1 Close apposition

Lekakou and Szendrői (2012) build on the observation that DS shows certain similarities to close apposition of nominals, illustrated in (50) for English and (51) for Greek.

(50)

a.

**Table:** 

*my friend the physicist*

b.

**Table:** 

*the actor Tom Hiddleston*

(51)

**Table:** 

Greek			
*o*	*aetos*	*to*	*puli*
the	eagle	the	bird
‘the eagle that is a bird’ (not the *aetos* that refers to a kite)			
(Lekakou and Szendrői 2012: 108)			

In close apposition, two DPs form one phonological phrase, and are linked as in (52) for Lekakou and Szendrői (2012).

(52)

**Table:** 

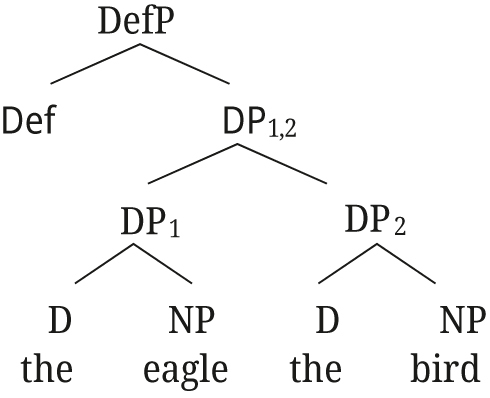

Semantically, DP_1,2_ is the intersection of the two DPs, established by Referential role identification between the two DPs (see Lekakou and Szendrői [2012] for details on the semantics). For the example in (52), DP_1,2_ refers to the entity that has both the property of being named *aetos* and that of being a bird. Lekakou and Szendrői (2012) then show how the same structure can account for the properties of polydefinites as well, if we assume that DP2 can have an elided NP and an adjective, as in (53).

(53)

**Table:** 

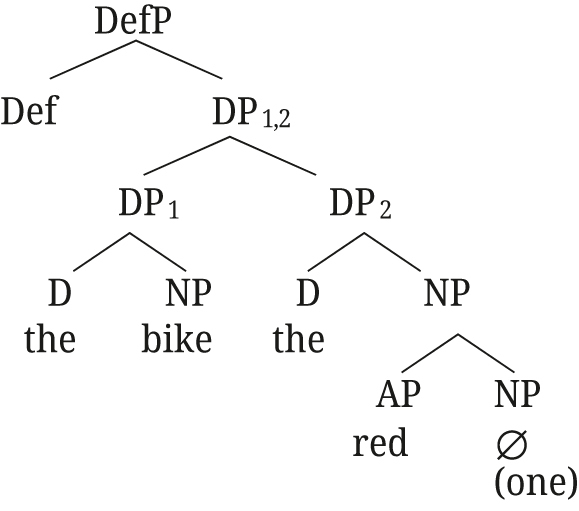

For monadic DPs (without DS), Lekakou and Szendrői (2012) propose the structure in (54), with a simple adjunction of AP to NP.

(54)

**Table:** 

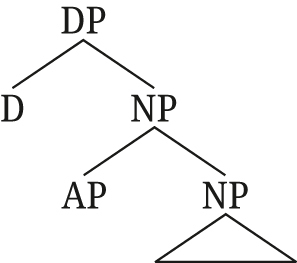

The structure proposed for DS by Lekakou and Szendrői (2012) naturally accounts for the presence of two determiners, as well as the restrictive reading of the adjective in polydefinite phrases: DP_1,2_ refers to an entity that is restricted to a subset by both properties of being a bike, and being red (unlike the adjunction structure in (54)). Furthermore, the structure in (53) explains the freedom in word order for polydefinites: DP1 and DP2 can easily be switched in this structure, whereas that is not the case for the simple adnominal adjective as in (54).

Turning to Rukiga, close apposition here too requires both DPs to appear with an augment, as shown in (55). Omitting the augment on either DP would be ungrammatical (note that names like Kato in (56) never show an augment).

(55)

**Table:** 

Naareet‘ [*(é)kit‘ *(é)nkoni] n‘ [*(é)kit’*(á)máarwa].					
N-aa-reet-a	e-ki-ti	e-n-koni	na	e-ki-ti	a-ma-arwa
1sg.sm-n.pst-bring-fv	aug-7-stick	aug-9-stick	and	aug-7-stick	aug-6-beer
‘I have brought the walking stick and the beer.’

(56)

**Table:** 

*W-aa-shashur-a*	*[Kat’*	*(ó)-mu-baizi]	*atári*	*[Kat’*	*(ó)-mu-híngi].
2sg.sm-n.pst-pay-fv	1.Kato	aug-1-carpenter	not	1.Kato	aug-1-farmer
‘You paid Kato the carpenter, not Kato the farmer.’					

The underlying structures for Rukiga along Lekakou and Szendrői’s (2012) analysis would thus be as in (57) for close apposition, and (58) for the [+A] adjective.15For the moment we remain agnostic about DefP in Rukiga.

(57)

**Table:** 

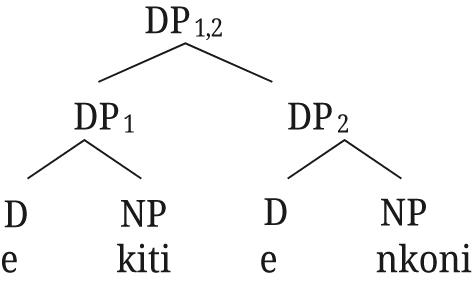

(58)

**Table:** 

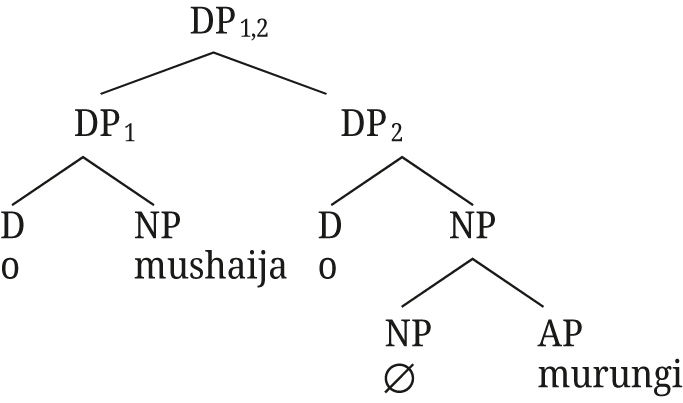

The symmetric structure of DP1 + DP2 makes (at least) the following predictions:

1. The prosody should remain the same, whether the order is DP1 > DP2 or DP2 > DP1;
2. Either DP should be able to head the construction and determine agreement outside the phrase.

The first prediction is borne out for Greek (59), but the situation in Rukiga is a bit more complicated.16It is worth noting, however, that there has been no careful investigation of the prosody of close apposition and DS in Greek. Impressionistically, a variety of intonational contours are possible in those constructions, and they seem to have an effect on interpretation. Therefore, further investigation might reveal interesting prosodic differences. DP1-DP2 always forms one intonation phrase in the restrictive interpretation, while the order DP2-DP1 can be phrased differently depending on the interpretation and the type of modifier. Possessives show an optional prosodic break, but prenominal adjectives require a break for some but not all speakers (60) (this differs for other modifiers in ways that are not entirely clear yet).

(59)

**Table:** 

Greek

a.

**Table:** 

*to*	*kokino*	*(to)*	*podilato*
the.n.sg	red.n.sg	the.n.sg	bike.n.sg

b.

**Table:** 

*to*	*podilato*	*(to)	*kokino*
‘the red bike’			
(Kolliakou 2004: 264)

(60)

**Table:** 

Rukiga

a.

**Table:** 

*e-bi-muri*	*(é-)bi-hángo*
aug-8-flowers	aug-8-big

b.

**Table:** 

*e-bi-hángo* ^*%*^ *(,)*	*(e-)bi-muri
‘big flowers’/‘big ones, flowers’	
(Asiimwe 2014: 257–258)

The second prediction is tricky: In noun-modifier combinations both DPs have the same ϕ features, making it impossible to see which of the two determines agreement. In traditional close apposition, however, DP1 and DP2 can differ in ϕ features. In Greek, the predicate can indeed agree with either DP, as seen in (61), although there is speaker variation in the agreement possibilities in examples like (61): we consulted three native speakers of Greek, and while one speaker accepted (61), two speakers only found agreement with DP1 grammatical.

(61)

**Table:** 

Greek

a.

**Table:** 

O	*aetos*	*to*	*puli*	*ine*	*megaloprepos/megaloprepo.*
the.m	eagle.m	the.n	bird.n	is	majestic.m/majestic.n

b.

**Table:** 

*To*	*puli*	*o*	*aetos*	*ine*	*megaloprepos/megaloprepo.*
the.n	bird.n	the.m	eagle.m	is	majestic.m/majestic.n
‘The eagle that is a bird is majestic.’					
(Lekakou and Szendrői 2012: 114)

In Rukiga, DP1 always determines agreement in close apposition (in line with Burton-Roberts 1975), illustrated in (62) and (63).

(62)

**Table:** 

Rukiga				
*[O-mw-ana*	*é-ki-handa]*	*a-ryá-sîng-a*	/	*ki-ryá-sîng-a.
aug-1-child	aug-7-fearless	1sm-fut-win-fv	/	7sm-fut-win-fv
‘The fearless child will win.’

(63)

**Table:** 

*[E-ki-handa*	*Rób]*	*ky-á-tu-sîng-a*	/	*y-á-tu-sîng-a.
aug-7-fearless	1.Rob	7sm-pst-1pl.om-win-fv	/	1sm-pst-1pl.om-win-fv
‘The fearless Rob has won from us.’				

Hence, Lekakou and Szendrői’s (2012) analysis of DS as close apposition, while initially looking promising, does not account for crucial aspects of the construction in Rukiga. Note that the agreement facts argue against the close apposition analysis in (55) for actual close apposition, but do not preclude an analysis of DP+modifier as such, as a reviewer points out. Nevertheless, missing that parallel is clearly undesirable.

Additionally, there are conceptual problems with the proposed structure: A more general theoretical problem with Lekakou and Szendrői’s (2012) analysis is that it does away with endocentricity (one head per phrase). Such a move potentially predicts the existence of structures like VP – VP, AP – AP, and so forth, with the risk of overgeneration. Burton-Roberts (1975) argues against ‘double endocentricity’ (two heads) of close apposition on the basis of referentiality (see also extended discussion in Acuña-Fariña 2016). In close apposition, it cannot be the case that both nouns are referential and corefer as in ‘*the King of Pop Michael Jackson’ (only possible as loose apposition), because if “such NPs were acceptable, we should be faced with the odd situation in which, each ‘constituent’ being coreferential with the other, the supposed superordinate entity would be coreferential with each and every one of its constituents, which inherently precludes it from being the superordinate entity and as such it would be totally redundant” (Burton-Roberts 1975: 396–397, via Acuña-Fariña 2016). One DP is hence the head, and the other functions as a predicate (Doron 1994). Furthermore, it is unclear how the computational system can distinguish between an adjunction and a close apposition structure for a derivation like (51). We therefore conclude that the analysis of DS as close apposition is suboptimal for the Rukiga facts and in general.

It is important to note here that Lekakou and Szendrői’s (2012) analysis for determiner spreading involves *close* apposition, not *loose* apposition. Heringa (2011: 3) describes the two types as follows: Close apposition “is restrictive. The two elements in this case are both required to describe their extralinguistic referent together. Loose apposition, on the other hand, is non-restrictive. One of the elements alone gives a unique description of the extralinguistic referent. The other just adds some extra information on that referent.” The difference can be seen in the minimal pair in (64), where the close apposition in (64a) does not have any prosodic marking, and entails that I have multiple sisters and restrict reference to the one named Hilde: both ‘sister’ and ‘Hilde’ are necessary to identify the intended referent. The loose apposition in (64b), on the other hand, is prosodically marked (by a possible preceding but at least following pause, and possible intonational cues such as a lower pitch; see i.a. Acuña-Fariña 1996; Dehé 2007), and entails that I only have one sister: the noun ‘sister’ and proper noun ‘Hilde’ individually refer to the same intended referent.

(64)

a.

**Table:** 

*My sister Hilde is a police officer.*	[close apposition]

b.

**Table:** 

*My sister, Hilde, is a police officer.*	[loose apposition]

Structurally, in loose apposition, the second DP is adjoined to the first [_DP1_ [DP2] [_DP1_ [D NP]]] (e.g., Potts 2005, going back to Jackendoff 1977). This adjunction to DP is what we propose for Rukiga loose apposition in Section 4.3.2., which is different in both form and interpretation from the close apposition illustrated above.17
Gambarage (2019) proposes an apposition structure for augments on modifiers in Nata, but from the tree structures he provides (i. and ii.) it is unclear whether close or loose apposition is intended. He describes that the presence of the augment on Nata modifiers is “to create DPs that can pick out a subset from the set referred to by the first DP” (Gambarage 2019: 140), i.e., the semantic effect we describe for Rukiga, but then proceeds to provide the translation as in Example (iii).

(i)

**Table:** 

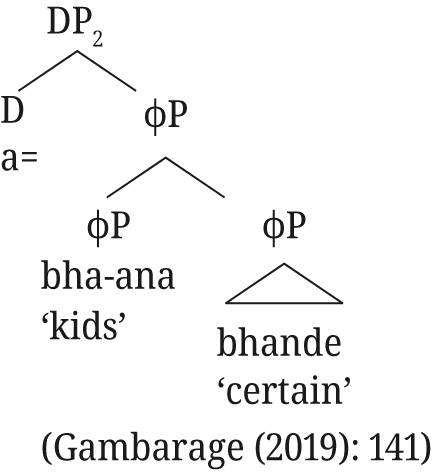

(ii)

**Table:** 

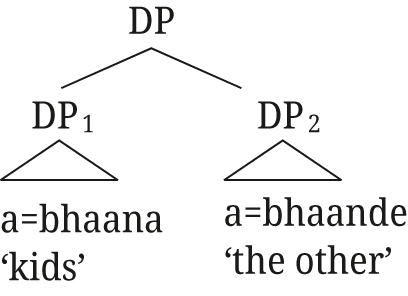

(iii)

**Table:** 

*o=mu-kári*	*o=mo-nyíini*	*a-ka-hét-a*	*ha-nọ.*
aug-1-woman	aug-1-wise	1sm-pst-pass-fv	16-dem.prox
lit. ‘The woman, the wise (one) passed here.’			
‘The wise woman passed here.’			
(Gambarage (2019): 140, glosses adapted)

We conclude, first, that Rukiga and Greek augment/determiner spreading is not loose apposition (see Alexiadou 2014: 34–35); and second, that the endocentricity in Rukiga (as seen in agreement) argues against an analysis as close apposition plus ellipsis. Close apposition and DS most likely constitute different phenomena (a point also made by Alexiadou 2014 for Greek, *contra* Lekakou and Szendrői). Lacking further data on close apposition in Rukiga, we leave its analysis as a topic for further research, but we assume that it will be different from the analysis of augment spreading to be developed in this article.

#### 4.2.2 Reduced relative clause

An alternative analysis of Greek DS builds on the observation that modification in the context of multiple determiners has the syntactic and semantic characteristics of indirect modification adjectives in Cinque’s (2010) terminology. Therefore, a reduced relative clause structure is natural, and has been proposed for Greek DS (Alexiadou 2014; Alexiadou and Wilder 1998).18There exist analyses of Greek that account for these observations without postulating a relative clause structure. For example, Panagiotidis and Marinis (2011) argue for a DP predicational structure in the case of DS in Greek. However, one of the main arguments that Panagiotidis and Marinis (2011) provide against the relative clause analysis is the lack of clear evidence in Greek for a CP layer with adjectives (and indeed DS is not possible with full relatives in Greek). Since augment spreading in Rukiga is possible with full relative clauses, the arguments against a CP layer do not hold in this case, which is why we focus here on Alexiadou and Wilder’s (1998) analysis. This type of analysis has also been applied to similar phenomena in Maltese (Winchester 2019) and the Nilotic language Kipsigis (Kouneli 2019).

A key assumption in these analyses is that relative clauses (at least restrictive ones in the context of DS) receive a raising analysis along the lines of Kayne (1994). More specifically, Alexiadou and Wilder (1998) propose the structure in (65) for the polydefinite DP: a determiner D, occupied by the definite article, takes a CP (or small clause SC) as a complement, which contains a DP (including an article) as the subject of an adjectival predicate. Then, this DP subject moves to SpecDP, which has been argued to be an A′-position with information structure effects in Greek on independent grounds (Horrocks and Stavrou 1987).

(65)

**Table:** 

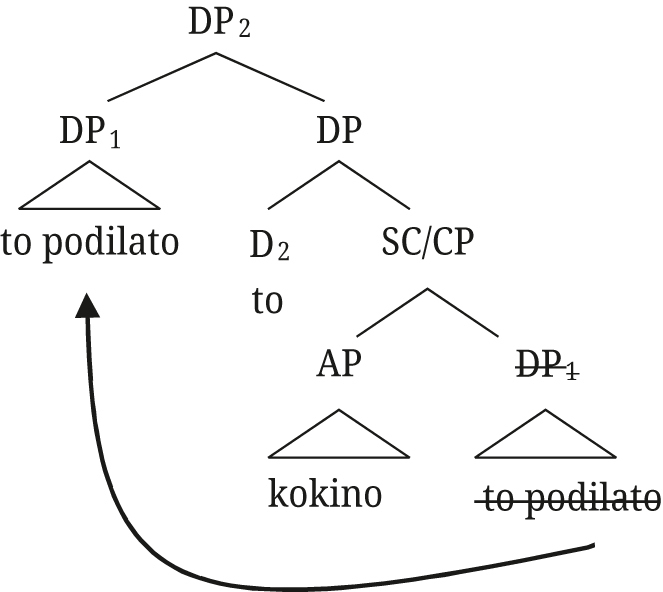

The structure in (65) derives the order in which the adjective follows the noun in Greek, but additional assumptions are needed for the pre-nominal orders. Alexiadou and Wilder (1998) propose different types of movements within the DP to derive these orders, but these are not always motivated. We leave aside the details of the Greek derivations for now, and discuss first how the main elements of the analysis fare with the Rukiga data.

The relative clause structure can straightforwardly extend to post-nominal [+A] modifiers in Rukiga: the derivation in (66) is exactly the same as the one in (65) for Greek, with the augment in the D position occupied by the article in Greek.

(66)

**Table:** 

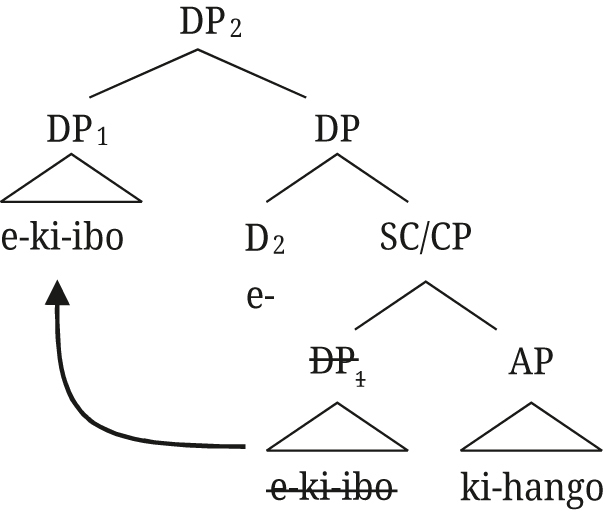

This analysis has a number of advantages for both Greek and Rukiga, apart from the fact that it accounts for the appearance of two determiners. The most important one is that it provides a straightforward explanation for why adjectives in DS in Greek and augmented modifiers in Rukiga pattern with Cinque’s (2010) indirect modification adjectives (which, as a reminder, are analyzed as reduced relative clauses) and receive a restrictive interpretation. This property follows automatically from the fact that additional determiners in both languages are only possible when a relative clause structure, as in (65), is present. This structure can also be extended to explain the presence of the augment not only on adjectives, but also on relative clauses in Rukiga (we return to Greek relatives in Section 4.4).

We conclude that the relative clause analysis along the lines of Alexiadou and Wilder (1998) is more promising in accounting for the distribution of the augment on modifiers in Rukiga. In the next section, we present a complete analysis of the Rukiga data, including a discussion of pre-nominal modifiers and further predictions of our analysis.

### 4.3 An analysis of Rukiga [+A]/[−A] modifiers

Before proceeding to our analysis of the Rukiga augment on modifiers, it is worth summarizing the patterns in Rukiga once more. In the simple case, modifiers are augmentless, and they appear post-nominally by default. When modifiers appear with an augment, on the other hand, they can appear pre- or post-nominally: without a prosodic break, they are associated with a restrictive interpretation, and with a prosodic break they form loose appositions. The augment occurs with a wide range of nominal modifiers: adjectives, relative clauses, possessives, and certain quantifiers. We choose here to focus on adjectives and relative clauses, and we leave the analysis of possessives and quantifiers as a topic for further research. There are two reasons for this choice. First, to analyze possessives and quantifiers in Rukiga, a more detailed investigation of their syntax and semantics is needed, one that is beyond the scope of this article. Second, adjectives and relative clauses are the modifiers that have featured in previous theoretical discussions of DS, making the connection and comparison of the Rukiga data to the phenomenon in other languages more straightforward.

In the rest of this section, we present our analysis for augmentless modifiers in 4.3.1, before analyzing augmented modifiers in 4.3.2. We argue in a nutshell that a different syntactic structure is involved in these two types of modifiers. We also discuss a third possible syntactic structure, one associated with augmented modifiers that involves a clear prosodic break between the noun and the modifier.

#### 4.3.1 Augmentless (direct modification) modifiers [−A]

Starting with the structure of a (augment-)Noun-Adjective phrase, as in (67), following standard practice we assume that the adjective is an adjunct to the NP, as shown in (68).19While we adopt a simple adjunction structure here, it should be noted that the data are also compatible with theories in which the adjective is generated in the specifier position of a dedicated functional projection, as is the cartographic tradition (Cinque 2005, 2010 among others). If such a structure is adopted, an additional mechanism is needed to derive the noun-initial order (see Cinque 2005 for possible movement steps in the DP).

(67)

**Table:** 

*e-ki-ibo*	*ki-hângo*
aug-7-basket	7-big
‘a/the big basket’

(68)

**Table:** 

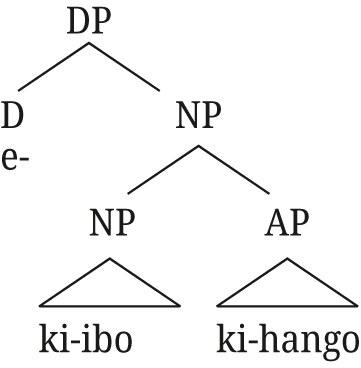

As for augmentless relative clauses, we assume that they also adjoin to the noun that they modify, with the augment that appears on the noun occupying the D position, as in (68). Nevertheless, it should be noted that at this stage, the Rukiga data at hand are compatible with a range of analyses for relative clauses.

#### 4.3.2 Augmented modifiers [+A]

We turn next to the analysis of augmented modifiers. As foreshadowed in Section 4.2, we believe that augmented adjectives and augmented relative clauses underlyingly have the same structure. The structure is the one assumed in raising analyses of relative clauses (Bianchi 1999, 2000; de Vries 2002 a.o.; Kayne 1994), where a D head takes a clausal (usually CP) complement. In Rukiga, this D head corresponds to the augment. What we see as the head noun of the relative clause originates as a DP_1_ (which already includes the augment) in the CP complement of D_2_. Following Alexiadou and Wilder’s (1998) analysis of Greek, we propose that this DP_1_ moves to the specifier position of the external D_2_.

In Alexiadou and Wilder’s (1998) analysis of DS in Greek, movement to SpecDP is motivated by independent evidence that SpecDP is an A-bar position with information structure effects in the language (Horrocks and Stavrou 1987). While further research is needed to determine the A versus A-bar status of the position in Rukiga, for now we have to stipulate movement to SpecDP_2_. We can think of this as a movement trigger on D_2_ associated with a feature [+rel], for example, raising the relativized DP out.

In (70), we provide a complete derivation for the augmented post-nominal relative clause in (69): the DP subject of the verb inside the relative clause (which already includes the augment) moves to the specifier of an external D head (=augment).

(69)

**Table:** 

*e-i-táági*	*é-rí-ri*	*hare*
aug-5-branch	aug-5rm-be	far
‘the branch that is far’

(70)

**Table:** 

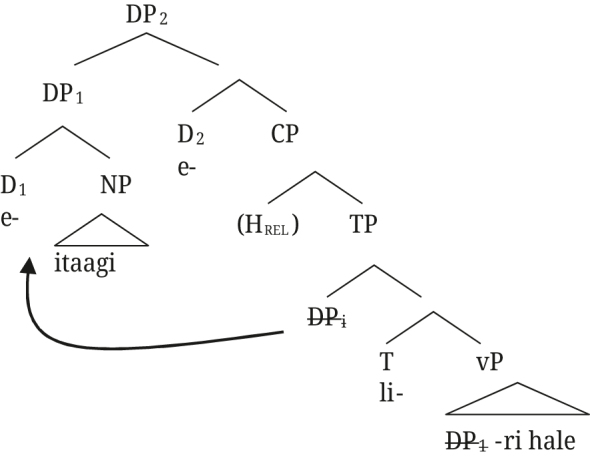

In (72) below, we provide the derivation for an augmented adjective, following Alexiadou and Wilder (1998) in assuming movement of the AP to SpecCP to form the relative clause, and movement of DP_1_ to SpecDP_2_, as above.20We remain agnostic about the amount of functional structure between SC/PrP and C. It may be that these clauses are tenseless or do not show vP, for example, but this does not affect our proposal. See also the discussion in Section 4.4.

(71)

**Table:** 

*e-kí-ibo*	*é-ki-hângo*
aug-7-basket	aug-7-big
‘a/the big basket’

(72)

**Table:** 

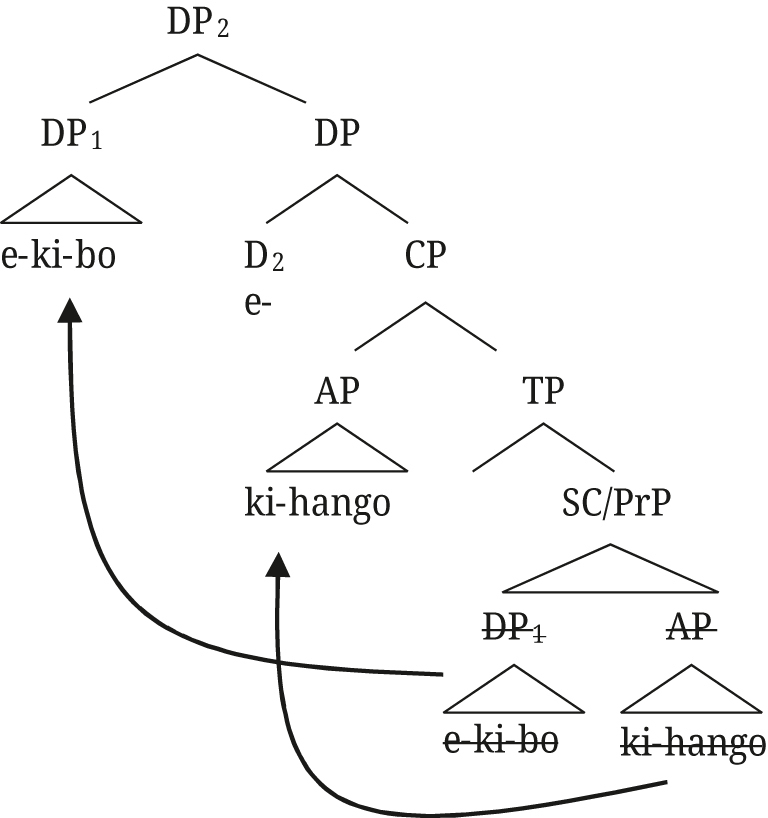

Note that nothing in the above structures requires that the DP subject of the CP is a single noun with an augment. This has at least two consequences. First, recursion is possible: for example, the whole DP_2_ in (72) could be the subject of another adjective, resulting in two [+A] adjectives following the noun. Second, any well-formed DP_1_ in the language could in principle be in subject position, which means that a DP containing one or more adjunct-adjectives (i.e., augmentless adjectives as in (67) above) could also occupy the subject position. Because DP_1_ then moves to SpecDP_2_, this makes certain predictions about the possible combinations of augmented and augmentless modifiers in a single DP. More specifically, it is predicted that augmentless modifiers must appear closer to the noun, with the augmented modifiers appearing further away. In other words, our analysis predicts that in noun-initial orders, once an augmented modifier is present, all modifiers to its right (linearly) must also have an augment. This prediction is borne out. We summarize the possibilities in (73): all adjectives can be [+A] as in (73a) or [−A] as in (73b), but if a [+A] and [−A] adjective co-occur, the [+A] adjective must follow the [−A] adjective, as the contrast between (73c) and (73d) shows.21Greek DS is subject to the same restriction (cf. Lekakou and Szendrői 2012: Section 3.1).

(73)

a.

**Table:** 

*e-bi-muri*	*é-bi-hángo*	*é-bi-rúngi*
aug-8-flower	aug-8-big	aug-8-good

b.

**Table:** 

*e-bi-muri*	*bi-hângo*	*bi-rungi*
aug-8-flower	8-big	8-good

c.

**Table:** 

*e-bi-muri*	*bi-hángo*	*é-bi-rungi*
aug-8-flower	8-big	aug-8-good

d.

**Table:** 

*e-bi-muri	*é-bi-hângo*	*bi-rungi*
aug-8-flower	aug-8-big	aug-8-good
‘(the) big good flowers’

It is worth pointing out that recursion is also predicted by Lekakou and Szendrői’s (2012) analysis and is not a deciding factor between the close apposition and the relative clause analysis. However, the restriction in [+A]–[−A] combinations are accounted for in the relative clause approach pursued here, but do not follow from in Lekakou and Szendröi’s close apposition approach, as DP_2_ could equally contain two modifiers, and hence the ungrammatical ordering of [+A] before [−A] is wrongly predicted to be allowed.

So far we have provided an analysis of [+A] *post*-nominal adjectives and relative clauses, which receive a straightforward explanation in Alexiadou and Wilder’s (1998) framework. Next we turn to *pre*-nominal [+A] modifiers, as illustrated in (74).

(74)

**Table:** 

*e-ki-hángo*	*é-ki-ibo*
aug-7-big	aug-7-basket
‘a/the big basket’	

Our proposal for this type of example is that the underlying structure is the same as in (72) for post-nominal [+A] modifiers, the only difference being the spell-out of the higher or lower copy, as represented in (75). If the higher copy of DP_1_ is spelled out, the resulting order is N-A, and if the lower copy is spelled out, we obtain A-N order. In the absence of a prosodic break (for which see loose apposition below), the interpretation of the prenominal and postnominal (augmented) adjective are both restrictive, which is in line with the optionality of spell-out. Per Reinhart’s (1996, 2006 Interface Economy, however, the optionality predicts a difference in interpretation, which is of a pragmatic nature: in Rukiga, the preverbal modifier is said to “add an emphatic assertion to remove any doubt”.

(75)

**Table:** 

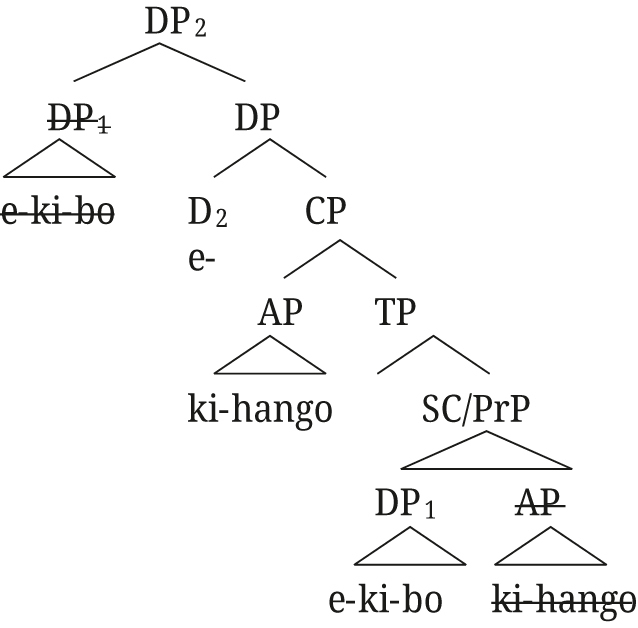

Finally, in many instances, a prosodic break is possible between the modifier and the DP. This different prosody clearly combines with a different interpretation, and, as we argue here, with a different underlying structure, too. As mentioned in Section 4.2.1, the phrases involving a prosodic break are loose apposition structures, where one DP is adjoined to another, as in (78). As expected, either order is acceptable, as illustrated in (76)–(77), and the interpretation is a sort of commentary on the first DP (see, among others, Koktová 1986), as is familiar from loose apposition crosslinguistically.

(76)

a.

**Table:** 

*e-nyungu,*	*e-m-pângo*
aug-9.pot	aug-9-big
‘the pot, the big one’

b.

**Table:** 

*e-m-pângo,*	*e-nyungu*
aug-9-big	aug-9.pot
‘the big one, the pot’

(77)

a.

**Table:** 

*e-ki-humi,*	*e-ky-áitu*
aug-7-granary	aug-7-poss.1pl
‘the granary ours’

b.

**Table:** 

*e-ky-áitu,*	*e-ki-humi*
aug-7-poss.1pl	aug-7-granary
‘ours, the granary’

(78)

**Table:** 

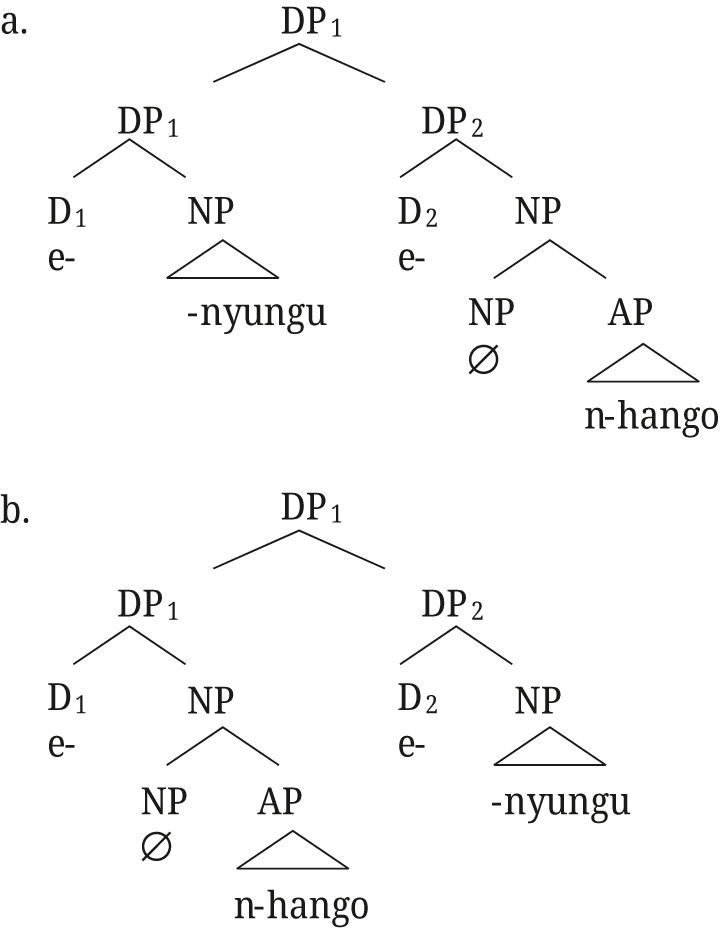

For prenominal relative clauses as in (79), we propose that this is in fact the only analysis. When a relative appears in prenominal position, it functions as a free relative (a DP), hence requiring an augment. It already gives a unique description of the referent. The noun following it, whether or not preceded by a prosodic break, is co-referential and interpreted as ‘already known or familiar’. Note that the restrictive interpretation may still be present, considering that DP1 is simply a restrictive relative clause with a null head noun (see (70) above).

(79)

**Table:** 

*e-í*	*ba-a-yómbek-ire*	*(,)*	*e-kanísa*
aug-9.rel.pro	2sm-f.pst-build-pfv		aug-9.church
‘the one that they built, the church’			

We now have a full account of the different orders of adjective and noun and the occurrence of the augment in Rukiga, summarized in Table 2.

**Table 2:** Analyses of adjective-noun combinations in Rukiga.

	[−A]	[+A] no break	[+A] break
Adj-N	*	Reduced relative	DP adjunction (loose apposition)
N-Adj	NP adjunction	Reduced relative	DP adjunction (loose apposition)

The same analyses ideally also hold for possessives and quantifiers. Considering the fundamental predicative relation at the basis of the reduced relative clause analysis, this implies that possessives and the quantifiers ‘few’, ‘many’, and ‘some’ may also function as predicates (which is uncontroversial for the former, but less accepted for the latter). We leave these implications for further research.

### 4.4 Some comments on the differences between Greek and Rukiga

Greek and Rukiga belong to different language families, and the properties of noun phrases in the two languages display a number of differences, which have been mentioned in passing so far. To name just a few: the default word order in the DP is different in the two languages, the Rukiga augment has different semantics from the Greek article, and Rukiga has a noun class system which does not look like the sex-based gender system of Greek. In this article, we have focused on the similarities that Greek and Rukiga display in the phenomenon of DS (as those were outlined in Section 4.1), and we believe that the broader differences in DP structure only strengthen the parallels drawn. In other words, DS in Greek and Rukiga looks very similar *despite* the many differences that we find in other aspects of the languages’ nominal syntax. Nevertheless, in Section 4.1, we also pointed out some small differences in the behavior of DS in the two languages. In this section, we briefly discuss those differences, and we provide possible explanations for the observed variation.

First, the distribution of the Greek article on nouns differs from that of the augment in Rukiga. For example, proper names never appear with the augment in Rukiga (see discussion in 4.1.1), but they require the article in Greek. In our analysis of DS, the crucial property that the augment and the article share is that they are both of category D (which can select for a CP complement). The semantics of D can, however, differ from language to language, explaining the differences in the distribution of the determiner on nouns. For example, it is standard practice to analyze both the Greek and the English article as elements of category D, but proper names in English differ from those in Greek in not tolerating the article. Thus, we believe that the different semantics of D and/or nouns across languages can (at least partially) account for distributional differences (see e.g., Matushansky 2006, 2008 for a semantic analysis of the variation in the use of the article with proper names).

The most important difference between Greek and Rukiga DS lies in the type of modifiers that can appear with a determiner. As was already mentioned in Section 4.1, DS in Greek is restricted to adjectives, numerals and some quantifiers, but it has a broader distribution in Rukiga, where it is possible with adjectives, quantifiers, possessives and full relative clauses. While a full explanation of this difference has to await further research, we discuss some analytical possibilities for possessives and relative clauses.

Possessives look quite different in the two languages. Possession in Greek is expressed via the genitive clitics of the personal pronouns (which never agree with any element in the DP), as shown in (80) for a first-person possessive. In Rukiga, on the other hand, there is no genitive case, and possessives share properties with adjectives; for example, they agree with the head noun in noun class, as shown in (81).

(80)

**Table:** 

*to*	*vivlio*	*mu*
the	book	1sg.gen
‘my book’

(81)

a.

**Table:** 

*a-ba-zaire*	*(á-)be*
aug-2-parent	aug-2.poss.1
‘his/her parents’

b.

**Table:** 

*e-n-te*	*(e-)ze*
aug-10-cow	aug-10.poss.1
‘his/her cows’

c.

**Table:** 

*e-n-te*	*(é-)z-angye*
aug-10-cow	aug-10-poss.1sg
‘my cows’

d.

**Table:** 

*e-ki-róoto*	*(é-)ky-aawe*
aug-7-dream	aug-7-poss.2sg
‘your dream’

Furthermore, Greek genitive possessive clitics cannot be used predicatively, as illustrated in (82). According to our analysis, only modifiers that can act as predicates can participate in DS, and we thus correctly predict that DS should be impossible with Greek possessives. Possessives in Rukiga, on the other hand, do appear in predicative position, as shown in (83), which explains why they can participate in DS.

(82)

**Table:** 

*To	*vivlio*	*ine*	*mu.*
the	book	is	1sg.gen
Intended: ‘The book is mine.’

(83)

**Table:** 

Ebimuri n’*(é)byangye.		
e-bi-muri	ni	e-by-angye
aug-8-flower	cop	aug-8-poss.1sg
‘The flowers are mine.’		

It is worth noting that Greek also has a ‘complex possession’ structure, in which the genitive clitic is accompanied by a dedicated possessive adjective *dhiko*; this construction has some similarities to possessives containing the adjective *own* in English, though the interpretation is not always identical (see Alexiadou 2005; Drachman 1991 for details). Interestingly, DS is possible with possessives if *dhiko* is present, as shown in (84).

(84)

**Table:** 

*to*	*dhiko*	*mu*	*(to)*	*vivlio*
the	own	1sg.gen	the	book
‘my own book’				

In the presence of *dhiko*, however, the genitive clitic has a possible host, and *dhiko*-clitic combinations are licit in predicative position, as shown in (85) (unlike bare clitics, see (82)). DS in possessives with *dhiko* is, thus, correctly predicted by our analysis. Furthermore, these data highlight that any differences between Greek and Rukiga in DS possibilities with possessives are not related to the semantics of possession in the two languages, but rather to the different syntax that possessives display (genitive clitics in Greek vs. adjectival possessives in Rukiga).

(85)

**Table:** 

*To*	*vivlio*	*ine*	*dhiko*	*mu.*
the	book	is	own	1sg.gen
‘The book is mine.’				

While the behavior of DS with possessives in Greek can be easily explained, it is less clear why DS is impossible with full relative clauses in Greek. Our analysis crucially relies on a relative clause structure, and we find DS with full relatives not only in Rukiga, but also in Kipsigis (Kouneli 2019). Therefore, we need to look for a Greek-specific property that would exclude DS with full relative clauses. We see two possibilities, which we outline below; we leave the choice between the two analyses as a topic for further research.

In our analysis, D takes a clausal complement: this is clearly a CP in the case of full relative clauses, but is most likely reduced (e.g., a PredP) in the case of adjectives, which generally lack tense (when used as nominal modifiers).22See Kouneli (2019: Ch. 5), among others, for evidence of a smaller size for reduced relatives in Kipsigis. In fact, reduced relatives behave differently from full relatives in most languages. For example, only reduced relatives can occupy a pre-nominal position in English. Thus, the difference between Greek and Rukiga could be due to selection: perhaps D in Rukiga can select for either CPs or PredPs, while D in Greek can only select for PredPs. Similar facts have been reported before in the literature on Semitic relative clauses: Siloni (1995) shows that reduced, but not full, relatives in Hebrew can be introduced by a determiner identical to the definite article, while Ouhalla (2004) argues that Amharic differs from Hebrew in allowing the article to introduce both reduced and full relatives.

An alternative explanation would appeal to the type of complementizer used in Greek relative clauses. More specifically, Greek full relative clauses are introduced by the complementizer *pu*, as shown in (86). This complementizer is otherwise used to introduce factive complements in the verbal domain, and Roussou (2010) has argued that *pu* has nominal properties and is inherently definite.

(86)

**Table:** 

*to*	*vivlio*	***pu***	*dhiavasa*	*hthes*
the	book	C	read.1sg	yesterday
‘the book that I read yesterday’				

It is therefore possible that CPs introduced by *pu* (i.e., full relative clauses) are incompatible with a definite D, which would explain why these CPs cannot be selected by D (and thus cannot participate in DS).23Note, however, that such an explanation also implies that Kayne’s (1994) raising analysis of relative clauses cannot be applied to Greek full relatives, at least not without further modifications.


## 5 Summary and discussion

We have shown on the basis of extensive tests that when Rukiga modifiers occur with an augment, their interpretation requires the presence of alternatives, that is, the referent of the modified DP must be interpreted as a subset of the larger set of DP entities, characterized by the modifying quality. This results in a restrictive reading of a [+A] relative clause, adjectives, possessives, and some quantifiers.

We have also demonstrated that the augment on Rukiga modifiers shows striking structural and interpretational parallels with Greek determiner spreading. The two languages differ in the breadth of application: where Greek shows determiner spreading on adjectives only, Rukiga also includes possessives, quantifiers, and relative clauses. We have shown how the reduced relative analysis proposed for Greek (Alexiadou 2014; Alexiadou and Wilder 1998) also works for Rukiga, making the correct predictions for the restrictive interpretation, ordering restrictions for N and modifier as well as [−A] and [+A] modifiers, and prosodic phrasing. This structural and interpretational analysis based on the comparison between Greek and Rukiga offers further perspectives in at least five areas.

First, under the proposed analysis, modifiers can take three different structures: one as NP adjunction, a second as a reduced relative clause, and a third as DP adjunction. The split between the first and the second provides support for theories of adjectival syntax that argue for two distinct structures for adjectival modification, one of which corresponds to a reduced relative clause (Cinque 2010 among others). In languages like Rukiga and Greek, morphology flags which structure a given DP has. However, various other Bantu languages do not have an augment at all, or do not use it in a similar way. For example, Kinyambo does not have an active augment, and adjectives can be used pronominally without requiring the augment to be present, as seen in (87). A further question is thus whether the underlying structures are the same in languages where D is not associated with segmental morphology.

(87)

**Table:** 

Kinyambo

a.

**Table:** 

Ba-kuru	bá-ka-jún-a.
2-mature	2sm-pst-help-fv
‘The mature ones helped.’

b.

**Table:** 

A-ba-kozi	ba-kúru	bá-ka-jún-a.
aug-2-worker	2-mature	2sm-pst-help-fv
‘The mature workers helped.’		
(Bickmore 1990: 14–15, via Downing and Marten 2019: 297)

Second, the analysis has consequences for debates about Universal 20, Greenberg’s (1963) universal about the order of demonstrative, numeral, and adjective with respect to the noun. In most generative theories aimed at deriving Universal 20, adjectives are treated as a uniform syntactic category cross-linguistically (e.g., Abels and Neeleman 2012; Cinque 2005). We have seen, however, that there are two types of adjectives, and indirect modification adjectives with a relative clause structure have a syntactic behavior that is different from what is usually assumed in studies on Universal 20 (which better corresponds to the structure for direct modification adjunct-type adjectives). This state of affairs highlights the need for caution when discussing adjectives in the context of Universal 20, as adjectives can have different syntax in different languages, making different predictions for word order variation. Furthermore, Bantu languages are known to show all sorts of deviations from the predicted word orders (Van de Velde 2022), and the answer to this state of affairs might lie in the syntax that indirect modification adjectives have in some Bantu languages (e.g., augmented adjectives in Rukiga).24See also Kouneli (2019: Chapter 5) for adjective-related problems for Universal 20 from Kipsigis, another East African language.
Van de Velde (2022) proposes the AMAR mechanism, which stands for Adnominal Modifier Apposition and Reintegration: a modifying expression can function as a referring expression with a null head, which is used in apposition to signal contrast (‘the giraffes, the BIG ones’). This appositional structure, with the pronominal modifier occurring further away from the head, may then undergo grammaticalization so that the (augmented, appositional) phrase is reintegrated into the DP. Such a scenario is very likely for Rukiga. As a consequence, the structure and syntactic status of the modifiers, and possibly the stage of the AMAR cycle that a language is in, should influence comparative studies of U20 such as Abels and Neeleman (2012) who mention Taylor’s data on Runyankore-Rukiga.

Third, the restrictive interpretation of the modifier requires the presence of alternatives, and suggests that the proposition is not true for these alternatives. Excluding some or all alternatives is known as ‘exclusive focus’ (cf. Rooth 1985, 1992, 1996). It remains to be seen, however, if the suggested exclusion is part of the semantics or the pragmatics associated with the augment/determiner spreading. If determiner spreading triggers *and excludes* alternatives, we predict that it would be infelicitous to assert that the proposition is true for the alternative referents too. However, Kolliakou (2004) shows for Greek that it is acceptable to do so, as shown in (88), where the presence of alternatives is required, but not their exclusion. It is the combination of determiner spreading and stress that brings about the exclusive focus reading in Greek, as shown in (89).

(88)

**Table:** 

O	Yannis	taise	ta	zoa.	I	mikres	i	gates	itan
the	Yannis	fed	the	animals	the	young	the	cats	were
pinasmenes,	opos	episis	ke	i	megales	(i	gates).		
hungry	as	also	and	the	big	the	cats		
‘Yannis fed the animals. The young cats were hungry, and so were the old ones.’

(89)

**Table:** 

O	Yannis	taise	ta	zoa.	I	MIKRES	i	gates	itan
the	Yannis	fed	the	animals	the	young	the	cats	were
pinasmenes,	^#^opos	episis	ke	i	megales	(i	gates).		
hungry	as	also	and	the	big	the	cats		
‘Yannis fed the animals. The young cats were hungry, ^#^and so were the old ones.’									
Kolliakou (2004)									

The same example replicated in Rukiga also shows that the alternatives are not necessarily excluded, considering that the continuation in (90) is acceptable in Rukiga, as is the sentence in (91).25We did find a difference in the continuing clause for the following examples. When the augment is present on the color-term relative clause, a following clause can indicate the inclusion of other books like black ones (as in ii) but is found awkward if the clause does not specify a subset, as in i. We have no analysis for this difference as yet.

(i)

**Table:** 

*N-aa-gur-á*	*e-ki-tabo*	*(* ^*#*^ *é)-ki-ríku-tukur-a.*		
1sg.sm-n.pst-buy-fv	aug-7-book	aug-7rm-prog-be.red-fv		
na	é-by-a	e-rangi	é-zí-ndi	n-áá-bí-gur-a.
and	aug-8-conn	aug-10.color	aug-10-other	1sg.sm-n.pst-8om-buy-fv
‘I have bought a red book, and (of) other colors I bought too.’

(ii)

**Table:** 

*N-aa-gur-á*	*e-ki-tabó*	*(é)-ki-ríku-tukur-a*.	
1sg.sm-n.pst-buy-fv	aug-7-book	aug-7rm-prog-be.red-fv	
hamwé	n’	é-ki-tabo	é-ki-ríkw-íragur-a.
and	and	aug-7-book	aug-7rm-prog-be.black-fv
‘I have bought a *red* book, and a black one I bought too.’

(90)

**Table:** 

*Yakóbo*	*ya-a-gabur-ir-a*	*é-n-yamáishwa.*			
1.Jacob	1sm-n.pst-feed-appl-fv	aug-10-animal			
Pusi	e-n-tó	za-a-ba	zi-ine	é-n-jara	
10.cat	aug-10-young	10sm-n.pst-be	10sm-have	aug-9-hunger	
na	púsi	é-n-kuru	za-a-ba	zi-ine	é-n-jara
and	10.cat	aug-10-old	10sm-n.pst-be	10sm-have	aug-9-hunger
‘Jacob fed the animals. The young cats were hungry, and also the old cats were hungry.’

(91)

**Table:** 

*Yaareeb’ éntéb’ (é)nungí neémbí nazó yáázíreeba.*			
y-aa-reeb-a	e-n-tebe	e-n-rungi	
1sm-n.pst-see-fv	aug-10-chairs	aug-10-good	
na	e-n-bi	na-zo	y-aa-zi-reeb-a
and	aug-10-bad	and-10.pro	1sm-n.pst-10om-see-fv
‘He saw good chairs, and bad ones he also saw.’			

This means that focus on a DP modifier is not inherently exclusive. Moreover, we may wonder whether focus on the sub-DP level exists at all. Consider the following: alternatives on the level of the modifier actually refer to alternative *referents* that have a different property. The alternative referents for ‘YOUNG cats’ is not ‘old’ but ‘old cats’. On the level of the modifier, the interpretation is only concerned with whether the indicated referent(s) are interpreted as restrictive, leaving room for other referents.

If true, this would facilitate the analysis of modified nouns and their function in the clausal information structure. In both Greek and Rukiga, DPs with and without determiner spreading/augmented modifiers can take various information-structural roles in the clause. We illustrate this for Rukiga: whether the DP functions as a left-peripheral topic, as in (92), or is focused, as in the cleft construction in (93), the augment on the modifier remains syntactically optional.

(92)

**Table:** 

*E-bi-kóp’*	*(é-)bi-hângo*	*n-aa-bi-teer-a=mu*	*á-ba-gyenyi.*
aug-8-cup	aug-8-big	1sg.sm-n.pst-8om-put-fv=18	aug-2-visitor
‘As for the big cups, I have served the visitors tea in them.’

(93)

**Table:** 

***E-bi-kópo***	***(é-)bi-hângo***	*ni-by-ó*	*n-aa-teer-a=mu*	*á-ba-gyenyi*.
aug-8-cup	aug-8-big	cop-8-rel.pro	1sg.sm-put-fv=18	aug-2-visitor
‘It is the big cups that I have served the visitors tea in.’				

Hence, there seems to be no correlation between “focus within the DP” (the restrictive reading of the augment) and focus in the clause: they are independent and all combinations occur.

Fourth, we can now return to the question of specificity, which earlier analyses of the augment proposed to be related to its presence (Asiimwe 2014). The intuition that the presence of the augment makes a reference specific follows automatically from the restriction: the augment specifies which subset out of a relevant set should be considered. Hence, no direct reference to specificity is needed, in our analysis.

Finally, our analysis connects the Rukiga augment to the linguistic debate of determiner spreading. It is thus worth discussing the implications of our analysis for the typology of the phenomenon. Alexiadou (2014), who provides the most in-depth typological study of determiner spreading, comes to the conclusion that multiple determiners in the DP show different characteristics cross-linguistically, with a unified analysis not being possible. She argues, however, that there are three broad types of analysis that can be applied to determiner spreading in a given language, summarized in (94).

(94)

a.

**Table:** 

[_DP_ [_CP_ [_IP_ DP AP]]]	reduced relative clause, e.g., Greek

b.

**Table:** 

[_DP_…[_FP_ AP [_DP_ ]]]	split-DP, e.g., Norwegian, Scandinavian

c.

**Table:** 

[_SC_ NP en AP]	spurious determiners, e.g., Hebrew
(Alexiadou 2014: 111)

The analyses in (94a) and (94b) are what Alexiadou (2014) calls the syntactic analyses of the phenomenon, since the additional determiners are linked to the presence of a specific syntactic structure: a relative clause in (94a) and a split DP (i.e., a DP with two D layers) in (94b). The former structure gives rise to unbounded determiner spreading (i.e., each modifier can be preceded by a determiner, irrespective of the number of modifiers), while the latter gives rise to the doubling phenomenon we find in Scandinavian, where there is a maximum limit of two determiners in the DP. The third type of analysis, illustrated in (94c) is what Alexiadou (2014) calls the morphological analysis: here, additional determiners are simply the morphological reflex of nominal concord for definiteness, which is the standard analysis for multiple definite articles in Semitic languages. In this type of analysis, we also find unbounded determiner spreading, but the claim is that there are no syntactic or semantic effects (e.g., a restrictive interpretation) associated with the additional determiners, which seem to be obligatory in those languages.

Having laid out the basics of Alexiadou’s (2014) typology, we conclude that Rukiga fits the pattern in (94a), with the phenomenon showing many similarities to the pattern of determiner spreading in Greek, as has been extensively discussed in this article. Therefore, Rukiga becomes the third language (after Maltese and Kipsigis; Kouneli 2019; Winchester 2019) to be added to the list of languages that fall under (94a) since the publication of Alexiadou’s (2014) typology. The analysis can thus be applied to phenomena from a wide range of language families, and it raises the question of whether it can be extended to other languages, including those that received a morphological analysis (94c) in Alexiadou’s (2014) terms (see Kouneli 2019: Ch. 5 for further discussion of this point). Nevertheless, even though the analysis is applicable to all these languages, we do observe differences in the type of modifier that triggers determiner spreading, as was already discussed. In Greek and Maltese, the phenomenon is mostly restricted to adjectives (and possibly numerals and some quantifiers), while in Rukiga and Kipsigis almost all nominal modifiers can appear with an additional determiner. Accounting for these cross-linguistic differences is an interesting avenue for further research.

## Abbreviations and symbols

We write all vowel length (phonemic and automatic) with 2 vowels. Orthographic <k> and <g> before [i], as well as <ky> and <gy> before other vowels, are pronounced [tʃ] and [dʒ], respectively. Although sometimes speakers pronounce [l], there is no <l> in orthography (instead <r> is used). Liaison between words is indicated by an apostrophe. When surface morphology is not transparent, a second line is added in examples, showing the underlying morphemes. High tones are indicated by an acute accent, low tones are unmarked. Numbers in glosses refer to noun classes unless followed by sg/pl [small caps] in which case they refer to person.

appl: applicative
aug: augment
C: complementizer
conn: connective
cop: copula
dem: demonstrative
DS: determiner spreading
f.pst: far past
fut: future tense
f: feminine
fv: final vowel
H: high tone
ipfv: imperfective aspect
m: masculine
n: neuter
ny: not yet
neg: negation
n.pst: near past
om: object marker
pfv: perfective aspect
pl: plural
poss: possessive
prog: progressive
PrP: Predicative Phrase
prs: present tense
pst: past
rel.pro: relative pronoun
rm: relative (subject) marker
SC: small clause
sg: singular
sm: subject marker

## Appendix

Table 3 summarizes the environments in which Rukiga nouns require, prohibit, or optionally allow the presence of the augment, to contextualize the discussion on modifiers in the main text (see also Asiimwe 2014, 2016; Gambarage 2019).

**Table 3:** The syntactic distribution of the augment on Rukiga nouns.

*Present*	*Absent*	*Optional*
Unmodified subjects	After quantifier *buri* ‘every’	Infinitives
Object of affirmative verb	Before wh word *ki* ‘which’	Object of negative verb
Object of preposition *na*	On predicative adjective	
Object of imperative verb	On most kinship terms	
Pronominal or prenominal modifier	On noun in the vocative	

There are two interesting environments in which nouns may vary in the presence of the augment. The first is infinitives, which are ambiguous between verbal and nominal. The nomino-verbal status of the infinitive becomes clear when examining the augment: An infinitive as a verb does not allow an augment (95a) while an infinitive that functions as a nominal requires an augment (95b).

(95) a.

**Table:**

*Ni-tu-teekateek’*	*(*ó)-ku-gyend-a*	*nyénsákáre.*
ipfv-1pl.sm-think	aug-15-go-fv	tomorrow
‘We expect to leave tomorrow.’		b.

**Table:**

**(O-)ku-tóngana*	*ni*	*ku-bi.*
aug-15-quarrel	cop	15-bad
‘To quarrel is bad.’		

A second environment is after a negative verb. According to Taylor (1985), the augment is not permitted to appear on a nominal after a negative verb, as is true for neighboring languages such as Luganda (Ashton et al. 1954; Hyman and Katamba 1993). However, it is observed that in some instances, (especially younger) Rukiga speakers use an augment on a nominal that immediately follows a negative verb. The presence of the augment in these cases seems to indicate the relevance of alternatives, comparable to the presence of the augment on modifiers.

(96)

**Table:**

^*%*^ *Tí-n-aa-gur-a*	*(é)-n-kaito.*
neg-1sg.sm-n.pst-buy-fv	aug-10-shoe
‘I have not bought shoes (with augment: I have bought something else).’			
